# Platelet C3G: a key player in vesicle exocytosis, spreading and clot retraction

**DOI:** 10.1007/s00018-023-05109-8

**Published:** 2024-02-12

**Authors:** Cristina Fernández-Infante, Luis Hernández-Cano, Óscar Herranz, Pablo Berrocal, Carmen Sicilia-Navarro, José Ramón González-Porras, José María Bastida, Almudena Porras, Carmen Guerrero

**Affiliations:** 1 Instituto de Biología Molecular y Celular del Cáncer (IMBCC), USAL-CSIC, Centro de Investigación del Cáncer, Campus Unamuno S/N, Salamanca, Spain; 2 Instituto de Investigación Biomédica de Salamanca (IBSAL), Salamanca, Spain; 3 Departamento de Medicina, Universidad de Salamanca, Salamanca, Spain; 4 Servicio de Hematología, Hospital Universitario de Salamanca, Salamanca, Spain; 5 Departamento de Bioquímica y Biología Molecular, Facultad de Farmacia, Universidad Complutense de Madrid, Ciudad Universitaria, Madrid, Spain; 6 Instituto de Investigación Sanitaria del Hospital Clínico San Carlos (IdISSC), Madrid, Spain

**Keywords:** RapGEF1, Granule secretion, Platelet spreading, Compound exocytosis, Actin polymerization, Secretome, Coagulation

## Abstract

C3G is a Rap1 GEF that plays a pivotal role in platelet-mediated processes such as angiogenesis, tumor growth, and metastasis by modulating the platelet secretome. Here, we explore the mechanisms through which C3G governs platelet secretion. For this, we utilized animal models featuring either overexpression or deletion of C3G in platelets, as well as PC12 cell clones expressing C3G mutants. We found that C3G specifically regulates α-granule secretion via PKCδ, but it does not affect δ-granules or lysosomes. C3G activated RalA through a GEF-dependent mechanism, facilitating vesicle docking, while interfering with the formation of the trans-SNARE complex, thereby restricting vesicle fusion. Furthermore, C3G promotes the formation of lamellipodia during platelet spreading on specific substrates by enhancing actin polymerization via Src and Rac1-Arp2/3 pathways, but not Rap1. Consequently, C3G deletion in platelets favored kiss-and-run exocytosis. C3G also controlled granule secretion in PC12 cells, including pore formation. Additionally, C3G-deficient platelets exhibited reduced phosphatidylserine exposure, resulting in decreased thrombin generation, which along with defective actin polymerization and spreading, led to impaired clot retraction. In summary, platelet C3G plays a dual role by facilitating platelet spreading and clot retraction through the promotion of outside-in signaling while concurrently downregulating α-granule secretion by restricting granule fusion.

**Supplementary Information:**

The online version contains supplementary material available at 10.1007/s00018-023-05109-8.

## Introduction

Rap1 GTPases are pivotal in platelet function, with previous knowledge attributing Rap1 activation solely to the guanine nucleotide exchange factor (GEF) RasGRP2 (CalDAG-GEFI) [1, 2]. In previous work, we have shown that another Rap1 GEF, C3G (RapGEF1), also plays an important role in platelet hemostatic function, in vitro and in vivo, regulating platelet activation and aggregation through a pathway involving PKC-Src-Rap1, among others [3, 4]. C3G also plays a role in platelet recovery after bone marrow depletion or thrombopoietin (TPO) stimulation [5, 6]. Moreover, C3G participates in platelet-mediated ischemia-induced angiogenesis and tumor metastasis, through the regulation of the release of angiogenic factors. Specifically, C3G overexpression in platelets induces the retention of proangiogenic factors within α-granules, while C3G deletion facilitates their release [6, 7]. However, despite the lower secretion, C3G transgenic platelets show increased spreading on poly-l-lysine by a Rap1-independent mechanism [7].

Vesicle secretion is a multi-step process involving: (i) transport of vesicles through cytoskeletal structures, controlled by Rab GTPases [8]; (ii) tethering/docking, in which vesicles establish contact with the plasma membrane (PM) and an initial association between v-SNAREs and t-SNAREs occurs, a process regulated by Ral GTPases through the exocyst complex, Munc13 and Munc18 proteins [9, 10]; (iii) priming, characterized by the interaction of v-SNAREs and t-SNAREs forming the trans-SNARE complex [10]; and (iv) fusion, in which the pore is formed and cargo is released [11].

Platelets undergo normal, full-collapse exocytosis, in which the vesicle membrane is incorporated into the PM during cargo release, increasing platelet surface area and thus, facilitating spreading [12]. A second type of exocytosis, termed kiss-and-run, has been described in double RalA/B KO platelets, in which, after a transient fusion of the vesicle with the target membrane and cargo release, the pore reseals and the vesicle is recycled, preventing platelet spreading [13, 14]. On the other hand, platelet α-granules can release their content individually (single exocytosis) or fused to other granules, a process known as compound exocytosis, depending on the level or time of stimulation [15].

Platelet secretion and spreading are sequential events involving the reorganization of the actin cytoskeleton and the participation of α-granules expressing the v-SNARE VAMP-7, which provide the extra membrane during spreading [12, 16]. These two processes are jointly regulated by two types of complexes: (i) the Arp2/3-VARP-VAMP-7 complex, where, in resting platelets, VARP sequesters Arp2/3 and VAMP-7 to prevent actin polymerization and VAMP-7 fusion with t-SNARE proteins, respectively [12]; (ii) and the trans-SNARE complex, where the SNARE domain of VAMPs engages the coiled-coil domain on t-SNAREs, thus driving membrane fusion [16, 17]. We and others have shown that C3G interacts with the subcortical actin cytoskeleton [18, 19]. In addition, C3G interacts with VAMP-7 in platelets [7]; therefore, C3G could play a role during platelet spreading by regulating actin dynamics and the complexes involved.

PKC proteins are main regulators of platelet secretion and spreading [20]. PKCα is the major PKC isoform in platelets and positively regulates platelet granule secretion through phosphorylation of SNARE complex proteins, such as SNAP23, syntaxin (Stx) 4, or Munc18 [20]. Other PKC isoforms, such as PKCβ, PKCδ and PKCθ also regulate granule secretion, but, in addition, have been associated with platelet spreading, where PKCδ regulates filopodia formation [20, 21]. Interestingly, PKC controls C3G activation in platelets [3, 4].

Platelet spreading requires the sequential formation of transient filopodia and sustained lamellipodia. The Rho GTPases are crucial regulators of this process. Cdc42 is commonly associated with filopodia formation, although its role in platelets is unclear [22, 23]. On the other hand, Rac1 promotes lamellipodia formation during platelet spreading through the regulation of WAVE-Arp2/3 [24]. A crosstalk between Rac1 and Rap1 in platelets has been described, where Rap1 regulates Rac1 activity, possibly through Vav and Tiam [25]. The Rap1-Rac1 axis also controls clot retraction [25], which occurs after spreading, when fibrin production by thrombin stabilizes the platelet plug [26]. In this process (secondary hemostasis), platelets provide a surface for the activation of coagulation complexes, through exposure of procoagulant phospholipids, such as phosphatidylserine (PS), and by serving as a reservoir of coagulation factors [27].

In this work, we have studied the role of C3G in platelet secretion, spreading and clot retraction using mouse models with differential expression of C3G in platelets, as well as in vitro approaches. We found that, during α-granule secretion, C3G modulates the formation of VARP-VAMP-7-Arp2/3 and trans-SNARE complexes, as well as Ral activation, in a Rap1-dependent manner. Furthermore, C3G promotes lamellipodia formation during platelet spreading through its participation in the Rac1-WAVE2-Arp2/3 pathway, independently of its GEF function. Finally, we present data indicating the participation of platelet C3G in the regulation of secondary hemostasis and clot retraction.

## Materials and methods

### Mouse models

The transgenic (tgC3G and tgC3GΔCat, expressed under the PF4 promoter) and conditional knockout (Rapgef1^flox/flox^;PF4-Cre^+/−^, hereinafter C3G-KO) mouse models for C3G used in this work have been described and characterized in previous works [3–7] and in Fig. S1A–C. WtC3G and wtC3GΔCat were the control mice for tgC3G and tgC3GΔCat mice, respectively, while C3G-wt (Rapgef1^flox/flox^; PF4-Cre^−/−^) was the control mice for the C3G-KO model. The SNAP23 knockout model was described in [28]. All mice used were 8–12 weeks old.

### Platelet purification and activation

Blood was obtained by cardiac puncture from mice anesthetized with 2% isoflurane (Vetfluorane^®^, Virbac), and collected into sodium citrate tubes (Sarstedt, Germany). Platelet rich plasma (PRP) was prepared from anticoagulated blood by double centrifugation at 100 × *g* for 4 and 5 min, respectively. Platelets were pelleted by centrifuging PRP at 1300 × *g* for 5 min and resuspended in Tyrode’s Hepes Buffer (134 mM NaCl, 0.34 mM Na_2_HPO_4_, 2.9 mM KCl, 12 mM NaHCO_3_, 20 mM HEPES [pH 7.4] and 5 mM glucose) and allowed to rest at RT for at least 30 min. After isolation, platelet number was equalized using Beckman Coulter Z2 particle counter.

Platelets were stimulated with the indicated agonists and conditions, depending on the assay. Platelet activation was determined by flow cytometry via measuring the high-affinity conformation of the integrin αIIbβ3 and the exposure of P-selectin after treatment with PKC inhibitors, as described [3].

### Analysis of platelet secretome

Secretome from thrombin-activated platelets (3 × 10^8^) was obtained as described [7]. Platelet releasates (20 μl out of 100 μl) were mixed with 8 μl 4X Laemmli Buffer and loaded into a 10% SDS–polyacrylamide gel. Proteins were detected by gel staining with Colloidal Blue (5% aluminum sulfate-[14–18]-hydrate, 0.02% Coomassie G-250, 20% ethanol, and 2% ortho-phosphoric acid [85%]).

The level of Tissue factor, Serpin E1, Endothelin, and PF4 in the releasate was measured using the Proteome Profiler Mouse Angiogenesis Array Kit (R&D Systems), following the manufacturer ´s instructions.

### Transmission electron microscopy

Platelet morphology and granularity were analyzed by transmission electron microscopy. Platelets were fixed with Fixation Buffer (2% glutaraldehyde, 1.6% PFA in 0.1 M Phosphate Buffer pH 7.4) for 30 min at RT, then washed with 0.1 M Phosphate Buffer (pH 7.4) and included in warm 2% agarose in dH_2_O at 50 °C for 5 min. After cooling, blocks were divided into pieces that were again incubated in Fixation Buffer for 30 min. After washing, the blocks were processed by the Microscopy Service of the University of Salamanca (Nucleus) for examination with a Tecnai Spirit Twin 120 kV transmission Electron microscope.

###  δ-Delta-Granule secretion

δ-Granule secretion was determined by flow cytometry by analyzing CD63 expression on the surface. Washed blood (30 μl), prepared as described [4], was incubated with anti-CD63-APC and anti-CD41-FITC antibodies (Table S2) for 15 min at RT in the presence of 0.5 U/ml thrombin and 2 mM Ca^2+^. CD41-positive (10,000) events were collected using a BD Accuri^TM^ C6 cytometer. Secretion of  δ-granules was also monitored by bioluminescence by measuring the amount of ATP released, using the commercial CellTiter Glo 2.0 kit (Promega G9241). PRP was adjusted to 9 × 10^6^ platelets and stimulated with different concentrations of thrombin (0.2, 0.5 and 1 U/ml) for 10 min at 37 °C under aggregating conditions. Platelets were centrifuged for 1 min at 650 × *g* and 15 µl of secretome were mixed with 15 µl of the reagent and added to opaque 384-well plates (Greiner) for 40 min at 37 °C. Bioluminescence was measured with an integration time of 0.75 s on a Tecan Infinite M200 Pro2 plate reader.

### Lysosome secretion

Lysosome secretion was monitored by measuring the release of β-hexosaminidase. Platelets (6 × 10^6^) were stimulated with different concentrations of thrombin (0.2, 0.5 and 1 U/ml) for 20 min at 37 °C. Platelet releasate was isolated after centrifuging for 1 min at 650 × *g*. Total sample controls were generated by lysing unstimulated platelets with 1% Triton X-100. Secretome (20 µl) was incubated with the same volume of 5 mM 4-NAG (4-Nitrophenyl *N*-acetyl-*β*-d-glucosaminidase) in Citrate Phosphate Buffer (80 mM Na_2_HPO_4_, 60 mM citric acid, pH 4.2) for 1 h at 37 °C in a 96-well plate (Corning). The reaction was stopped by adding 0.1 M NaOH and the absorbance was read at 405 nm on a Tecan Infinite M200 Pro2 plate reader.

### Platelet endocytosis

Platelets were incubated with 5 μM AF488-labeled fibrinogen (Molecular Probes, F13191) for different times (0, 5, 15, and 30 min) at 37 °C. Then platelets were fixed with 8% paraformaldehyde (PFA) and seeded on poly-l-lysine-treated coverslips. Attached platelets were permeabilized with 0.2% Triton X-100 and blocked with 1% BSA. After labeling with phalloidin-iFluor 647 (Table S2) to visualize actin filaments, preparations were mounted and images were obtained with a Leica TCS SP8 confocal microscope system using a 100x/1.4 Oil Plan-ApoChromat Ph1 immersion objective and an extra 12X digital zoom.

### Platelet spreading

To analyze platelet spreading, coverslips were coated with Poly-l-lysine hydrobromide (Sigma P6282), Type I Collagen (Stem cell 07001), CRP-XL (Peptide Synthetics), Fibrinogen (Sigma F3879), Fibronectin (Sigma F1141), Laminin (Roche 11,243,217,001) or vitronectin (Advanced BioMatrix 5051) o/n at 4 °C. Platelets were treated with inhibitors and agonists as indicated and added to coated coverslips previously blocked with 1% BSA for 1 h at RT. Then, they were allowed to spread at 37 °C at various times (0, 5, 15, and 30 min), fixed with 4% PFA, permeabilized with 0.2% Triton X-100, blocked with 1% bovine serum albumin (BSA) and stained with phalloidin-iFluor 488 (Table S2) to visualize the actin cytoskeleton. The images were obtained with a Leica TCS SP8 confocal microscope with a 100X objective and an extra 12X digital zoom. Platelet area was analyzed with ImageJ/FIJI software [29].

To monitor spreading in real time, 1 × 10^5^ platelets were seeded on ibiTreat-coated µ-Slide 8-well plates (Ibidi). Platelets were recorded for 5 min at 37 °C and 5% CO_2_ using differential interference contrast (DIC), to assess platelet morphology, with a Nikon Eclipse TE2000-E microscope coupled to a video camera Hamamatsu Orca-er and processed with Metamorph^®^ software. Images were acquired every 10 s and then stacked and processed using ImageJ/FIJI software [29].

### Determination of F/G actin ratio

Platelets (5 × 10^6^), pretreated or not with latrunculin A (10 µM), were activated with 0.2 U/ml thrombin and immediately lysed with an equal volume of Lysis Buffer (100 mM Tris–HCl pH 7.4, 10 mM EDTA, 2 mM MgCl_2_ and 2% Triton X-100) supplemented with protease and phosphatase inhibitors (2 mM Na_3_VO_4_, 5 mM NaF) and kept on ice for 10 min. Lysates were ultra-centrifuged at 100,000 × *g* for 1 h, at 4 °C to separate Triton-X-100-soluble G-actin from Triton-X-100-insoluble F-actin. Supernatant containing G-actin was collected, and the F-actin pellets were solubilized with an equal volume of 8 M urea. Fractions were separated by SDS-PAGE and immunoblotted. F-actin and G-actin were detected with anti-β-actin antibodies (Table S2).

### Determination of F/G actin ratio in spread platelets

Platelets (5 × 10^8^) were activated with thrombin (0.2 U/ml) and let spread for 30 min at 37 °C on coverslips pretreated with collagen-related peptide (CRP-XL) or fibrinogen and blocked with 1% BSA. After that, coverslips were washed with PBS to remove non-adhered platelets. Platelets were collected with trypsin and immediately lysed with Lysis Buffer and processed as described above.

### Confocal immunofluorescence microscopy

After stimulation, platelets were fixed with 8% PFA 1:1 (v/v) for 15 min at RT before being seeded onto glass coverslips pre-coated with poly-l-lysine for at least 50 min at RT. Attached platelets were washed twice with PBS and then permeabilized with 0.2% Triton X-100 for 10 min. After washing twice, platelets were blocked with 1% BSA o/n at 4 °C. Coverslips were incubated with the primary antibodies (Table S2) for 2 h at RT and washed twice again before incubating with secondary antibodies for 1 h at RT in darkness (Table S2). Phalloidin-iFluor 488 was used for actin detection. Coverslips were mounted with ProLongTM Diamond Antifade Mountant (ThermoFisher Scientific).

Images were captured using a Leica TCS SP8 confocal microscope system with a 100X/1.4 Oil Plan-ApoChromat Ph1 immersion objective and an extra 12X digital zoom. Images were processed with Leica LAS X Software using Lightning process, and analyzed with ImageJ/FIJI Software. Protein colocalization studies were performed using Coloc2 plug-in, analyzing Pearson’s correlation coefficient [7]. Protein distribution was analyzed using the Plot Profile plug-in (ImageJ/FIJI software).

### Quantification of F-actin levels by flow cytometry

Washed platelets, obtained by washing PRP with Tyrode’s Hepes Buffer, were stimulated with 0.2 and 1 U/ml thrombin and fixed with 2% PFA for 15 min at RT. After washing twice, platelets were permeabilized with 0.2% Triton X-100 and blocked with 1% BSA for 30 min. Platelets were stained with phalloidin-iFluor 488 for 30 min at RT and darkness. Mean Fluorescence Intensity (MFI) of actin-labeled platelets (1 × 10^4^) was analyzed on a BD Accuri™ C6 cytometer, identifying platelets according to their FSC/SSC features.

### FM™ 1–43 staining assay

To monitor the dynamics of platelet granule release, we used the dye FM™ 1–43 (*N*-(3-Triethylammoniumpropyl)-4-(4-(Dibutylamino) Styryl) Pyridinium Dibromide), hereinafter FM1-43, which has a high affinity for membranes. After staining, when vesicles undergo exocytosis in dye-free medium, FM1-43 molecules dissociate from the membrane, thus fluorescence is lost [30]. The FM1-43 signal only remains in recycled vesicles, i.e., in those undergoing kiss-and-run exocytosis, but is lost in full-collapse secretion [31, 32]. Platelets (1 × 10^7^) were incubated with 10 µM FM1-43 (Table S2) at 37 °C for 30 min. The excess of FM1-43 was removed by centrifuging at 1300 × *g* for 5 min. Stained platelets were then plated on a µ-Slide 8-well ibiTreat plate (Ibidi). Decrease in signal (dye diffusion) from synaptic vesicles was monitored by fluorescence imaging. Platelets were recorded every 1.3 s at 37 °C and 5% CO_2_ with a Leica TCS SP8 confocal microscope with a 100x/1.4 Oil Plan-ApoChromat Ph1 immersion objective and an extra 10X digital zoom.

Granule exocytosis analysis was performed by measuring intensities of the same granule tracked along successive frames. Two different secretion patterns were established: (i) kiss-and-run, when FM1-43 MFI is maintained over time (a subtle decrease was observed due to dye bleaching), indicating retention of FM1-43 in the vesicle; (ii) full-collapse exocytosis, in which FM1-43 MFI decreases due to rapid diffusion of the dye to the PM.

### Transient transfection of HEK293T cells

HEK293T cells were grown in Dulbecco’s modified Eagle’s medium (DMEM, Sigma-Aldrich) with 10% fetal bovine serum (FBS; Life Technologies), penicillin (100 U/ml), and streptomycin (100 μg/ml) (Life Technologies) at 37 °C and 5% CO_2_. Cells (1 × 10^6^) were transfected using polyethylenimine (PEI, Polisciences Inc., PEI:DNA ratio 2.5:1) with plasmids: pCEFLHA-C3G [34], pcDNA-HA-CrkL [34], RFP-Abi1, a gift from Dr. L. Machesky (Beatson, UK), pCDNA-CFP-C4-WAVE2 (generated in this work) and the Addgene plasmids: pEGFP-VAMP-7 (#42316), pEGFP-VAMP-8 (#42311), tdTomato-VASP-5 (#58141), pEGFP-VARP (#42312), pEGFP-SNAP23 (#101914), mEmerald-ARP3-N-12 (#54979), pEGFP-C3-Sec3 (#53755), pEGFP-C3-Exo70 (#53761) and mEmerald-WASP1-C-14 (#54314). After 48 h cells were lysed with MLB (20 mM Tris–HCl pH 7.5, 150 mM NaCl, 0.5% Triton X-100) supplemented with 1 mM Na_3_VO_4_, 25 mM NaF, 1 mM PMSF, 1X cOmplete protease inhibitor cocktail (Roche) for 10 min on ice.

### Western blot and immunoprecipitation

Platelet protein extracts for western blot were prepared by lysing cells directly in sample loading buffer, as described [33].

For immunoprecipitation, platelets from three mice were lysed in 100 μl of standard RIPA buffer [7] and immunocomplexes (from 80 μl lysate) were pulled-down using anti-C3G (G-4), anti-SNAP23 and anti-Arp2 (B-6) antibodies (Table S2). The remaining 20 μl were used as a loading control. HEK293T were lysed in MLB (see above) and immunocomplexes were pulled-down with anti-HA antibodies (Table S2). Complexes were purified with protein G agarose resin 4 rapid run (ABT). Antibodies used for western blot are detailed in Table S2.

### GTPase activation assays

The activated, GTP-bound forms, of Rac1, Cdc42, RhoA and RalA were pulled-down using GST-PAK-RBD (for Rac1 and Cdc42), GST-Rhoketin-RBD (for RhoA), both gifts from Dr. X.R. Bustelo (IBMCC, Salamanca, Spain) and GST-RalBP1 (for RalA), a gift from Dr. A.W. Poole (University Walk, Bristol, UK), immobilized on glutathione-sepharose beads. Platelets (2 × 10^8^) were stimulated with thrombin (0.2 or 1 U/ml) at RT for 1 min, except for Ral assays in which platelets were stimulated at 37 °C and under stirring conditions. After that, platelets were lysed for 10 min at 4 °C with the same volume of 2X RIPA Modified Buffer [4] or, in the case of RalA assays, with 2X Lysis Buffer (20% Glycerol, 2% NP-40, 400 mM NaCl, 50 mM HEPES pH 7.4, 20 mM MgCl_2_), supplemented with 20 µg of the different fusion proteins. The different GTPases were detected by western blot with specific antibodies (Table S2).

### Lentiviral production and transduction of PC12 cells

Lentiviral particles were produced by transient transfection of HEK293T cells, as described [34]. PC12 cells were grown in DMEM medium supplemented with 10% Horse Serum (Gibco), 5% FBS, 1% MEM Non-Essential Amino Acids (Gibco), penicillin (100 U/ml), and streptomycin (100 μg/ml) on plates previously coated with 50 µM Type I Collagen (StemCell) in 20 mM acetic acid. PC12 cells were infected with the recombinant virus pLenti-C-mEGFP-IRES-BSD, empty or expressing wild-type human C3G or hyperactive C3G-Y554H mutant [34], or with the pLVTHM-C3Gi construct to silence C3G expression [5] in the presence of polybrene (4 μg/ml) (hexadimethrine bromide, Sigma-Aldrich). Infected cells were selected with blasticidin (2 μg/ml; InvivoGen, added 24 h post-infection) for at least 4 weeks before analysis, or by FACS (GFP expression).

### Analysis of granule exocytosis of PC12 cells by TIRFM: Detection of single-vesicle exocytosis by NPY secretion, and pore formation by analysis of VAMP2-pHmScarlet

The above PC12 clones were nucleofected with 25 µg of NPY-td-Orange2 plasmid (Addgene #83497) or 30 µg of VAMP2-pHmScarlet plasmid (Addgene #166890). NPY (neuropeptide Y) is a vesicle marker and reporter of dense core granule exocytosis and release [35]. pHmScarlet is a pH-sensitive fluorescent protein that enables the simultaneous detection of vesicle docking and fusion [36]. 48 h post-transfection, 3 × 10^5^ cells were seeded at a density of 0.75 × 10^5^ cells/ml on 25-mm cover glasses (Deckgläser) previously coated with poly-l-lysine and type I collagen. After 72 h, the culture medium was replaced with Ringer Buffer (RB: 130 mM NaCl, 3 mM KCl, 5 mM CaCl_2_, 1.5 mM MgCl_2_, 10 mM glucose and 10 mM HEPES pH 7.4). Cover glasses were mounted into AttoFluor™ Cell Chambers (ThermoFisher) in a THUNDER Image Live Cell 12899 SP8 Leica inverted microscope system equipped with a 100X/1.47 Oil Plan-ApoChromat Ph1 immersion objective type TIRFM (Total Internal Reflection Fluorescence Microscopy) system. PC12 cells were stimulated by replacing the RB with high KCl Modified Ringer Buffer (MRB: 37 mM NaCl, 56 mM KCl, 5 mM CaCl_2_, 1.5 mM MgCl_2_, 10 mM glucose and 10 mM HEPES pH 7.4) and maintained at 37 °C.

All TIRFM images were acquired at the same intensity and the same grade of penetrance and were taken every 0.3 s (∼3 frames/sec) for 30 s.

We started studying the MFI before the exocytic event until it disappeared (4 frames earlier in the NPY secretion and 1 frame earlier in pore formation analysis). We normalized the MFI to the exocytic event, which is considered 100%. Then, we studied the kinetics of NPY secretion and the MFI of VAMP2-pHmScarlet. Normally the intensity value of the peak frame (exocytic event) decays rapidly to values lower than those of the first frames. We monitored the intensity manually (by drawing the ROIs and measuring the MFI in the different frames). The granule number, area of granules and the number of exocytic events were quantified with ImageJ/FIJI software [29].

### Analysis of PC12 secretion

PC12 cells (1.5 × 10^5^) were plated in 12-well plates (Corning). Next day, cells were starved for 5 h at 37 °C (DMEM + 2% Horse Serum + 1% FBS). Then, medium was replaced by MRB (see above) and cells were stimulated for 1 h at 37 °C. The medium was collected and centrifuged at 300 × *g* for 5 min at 4 °C to remove dead cells and debris. The protein in the medium was quantified by the Bradford assay [37].

### Clot retraction

PRP was adjusted to 5 × 10^8^ platelets/ml, which were subsequently stimulated with 1 U/ml thrombin to initiate coagulation. Clot retraction was monitored for 3 h at 37 °C, taking photographs at 30 min intervals. The clot area was analyzed with ImageJ/FIJI Software [29] and the retracted volume and clot weight were measured at the end of the experiment.

### Detection of phosphatidylserine (PS) exposure

Washed platelets, isolated from blood obtained by cardiac puncture and anticoagulated with citrate, were stimulated with 1 U/ml thrombin and with different concentrations of CRP-XL (2, 5 and 10 µg/ml), in the presence of anti-CD41-FITC antibody and Annexin V-APC (Table S2) at RT and darkness [38, 39]. After 15 min, platelets were washed with Binding Buffer (0.1 M HEPES [pH 7.4], 1.4 M NaCl, 25 mM CaCl_2_) and analyzed by flow cytometry, on a BD Accuri™ C6 cytometer, for Annexin-V labeling in 20000 CD41-positive events.

### Thrombin generation

Thrombin generation measurements were conducted by Calibrated Automated Thrombinography (CAT) [40]. PRP (60 µl) was mixed with 20 µl of different trigger solutions, denoted PPPLow (mixture of 1 pM TF and 4 pM phospholipids); MP reagent (4 pM phospholipids) or PRP reagent (1 pM TF), and subsequently coagulation was initiated by the addition of 20 µl FluCa buffer containing a 7-amino-4 methylcoumarin (AMC)-based fluorescent substrate (Z-Gly-Gly-Arg-AMC) and CaCl_2_.

All measurements were performed per duplicate. To correct for differences in plasma color, each plasma measurement was calibrated against the same plasma mixed with 20 μl thrombin calibrator (Thrombinoscope BV), also per duplicate. The fluorescence of AMC was measured using a Fluoroskan Ascent (Thermo Scientific, Waltham, MA, USA) equipped with a 390 nm excitation and 460 nm emission filter set. The Thrombinoscope BV software was used to calculate lag time, endogenous thrombin potential (ETP) or AUC (area under the curve), peak, and time to peak (ttPeak).

### Statistical analysis

Data have been represented as the mean ± SEM (Standard Error of the Mean) or the mean ± SD (Standard Deviation), as indicated in each figure. At least 2 independent experiments from each genotype (mice and cell lines) were performed. In fluorescence measurements (MFI), the data in each experiment was normalized against the control values, due to the high variability between independent experiments.

The Kolmogorov–Smirnov test was performed to determine if data fit into a normal distribution. To compare between two experimental groups, unpaired Student’s *t* test was computed when the data were normally distributed. The Mann Whitney’s *U* test was computed as a non-parametric procedure when the data were not normally distributed. Differences were considered statistically significant when p value was less than or equal to 0.05. Both statistical methods were performed using GraphPad Prism v8 and Sigma Plot v11.0 software.

## Results

### C3G regulates α-granule distribution and secretion. Role of PKCδ

**Fig. 1:**
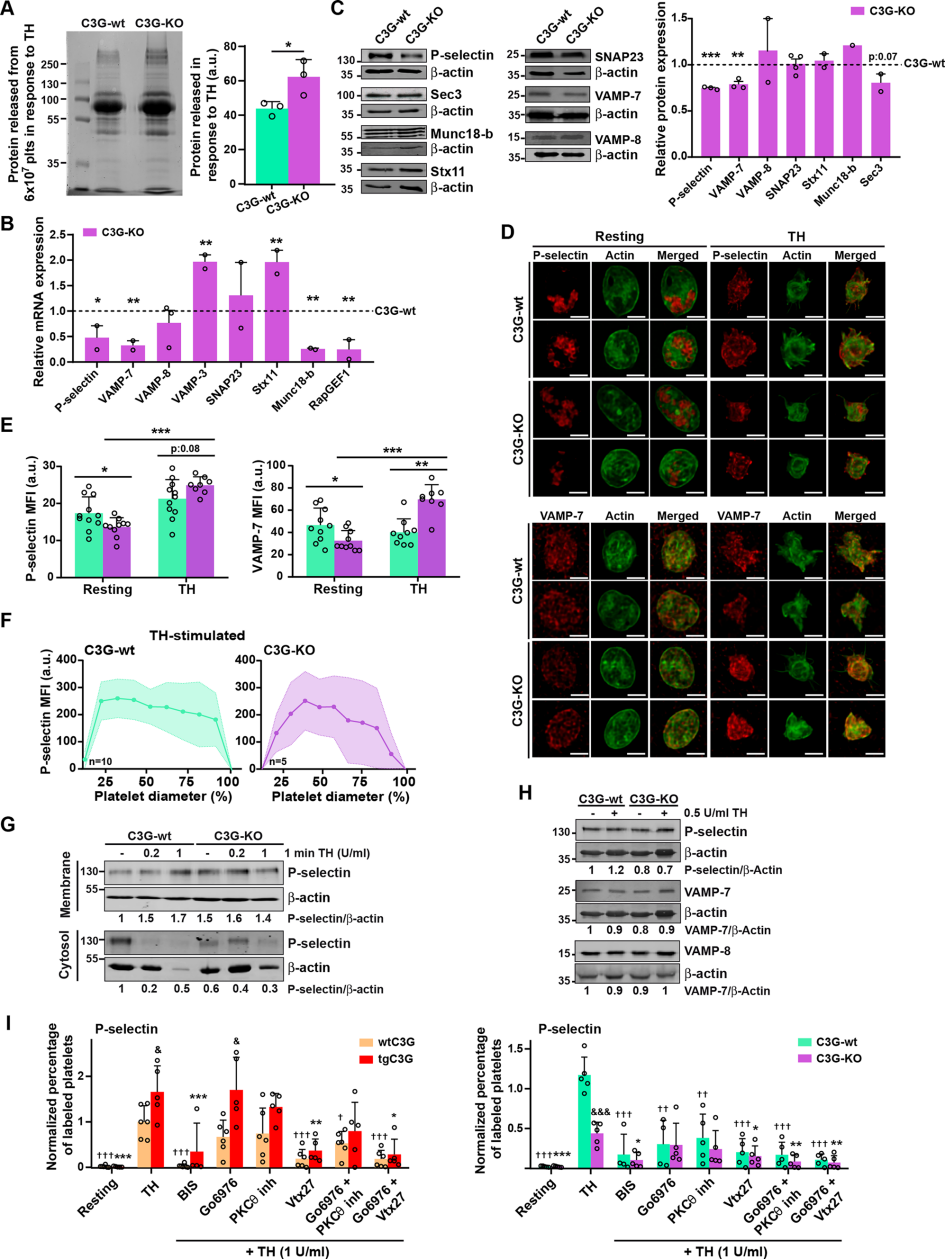
C3G controls α-granule secretion through PKCδ. **A** SDS-PAGE of secretome proteins from C3G-wt and C3G-KO 6 × 10^7^ platelets (plts) stimulated with 0.2 U/ml thrombin (TH) for 5 min at 1100 rpm and 37 °C. Proteins were visualized with Colloidal Blue Staining. Histogram represents the mean ± SD of the quantification of proteins in the releasate. **B** RT-qPCR analysis of the expression of the indicated genes in mRNA from C3G-KO and C3G-wt platelets using β-actin as housekeeping gene. C3G (*RapGEF1*) was used as control. **C** Western blot analysis of P-selectin, Sec3, Munc18-b, Syntaxin 11 (Stx11), SNAP23, VAMP-7 and VAMP-8 protein levels in lysates from C3G-wt and C3G-KO resting platelets. Bar graphs represent densitometric analysis of protein bands, relative to β-actin expression and normalized against wild-type platelets. **D** Representative immunofluorescence confocal microscopy images of C3G-wt and C3G-KO platelets stimulated with 0.5 U/ml thrombin (TH) for 1 min and labeled with antibodies anti-P-selectin + Cy3 (upper) or anti-VAMP-7 (H-55) + AF568 (lower) (red), and with phalloidin-iFluor 488 (green). Scale bar: 2 µm. **E** Bar graphs represent the mean ± SD of the MFI of P-selectin (left), or VAMP-7 (right) in resting platelets and in response to thrombin. **F** Line/scatter plots represent the mean ± SD of P-selectin distribution along the platelet diameter (MFI). **G** C3G absence alters P-selectin distribution in platelets. Western blot analysis of P-selectin levels in the membrane (upper) or cytosolic (lower) fractions from resting, 0.2 or 1 U/ml thrombin (TH)-stimulated C3G-wt and C3G-KO platelets. β-actin was used as loading control. Values of the P-selectin/α-actin ratio were normalized against those of resting C3G-wt platelets. **H** Western blot analysis of P-selectin, VAMP-7 and VAMP-8 levels in resting and 0.5 U/ml thrombin-stimulated C3G-wt and C3G-KO platelets. β-actin was used as loading control. Values are relative to β-actin expression and were normalized against resting C3G-wt platelets. *p ≤ 0.05, **p ≤ 0.01, ***p ≤ 0.001. **I** Washed blood from (left) wtC3G and tgC3G or (right) C3G-wt and C3G-KO mice was pre-treated with 1 µM Go6976 (an inhibitor of PKCα and β), 20 µM PKCθ inhibitor or 100 µM Vtx27 (wtC3G and tgC3G) or 2 µM Go6976, 40 µM PKCθ inhibitor or 200 µM Vtx27 (C3G-wt and C3G-KO) for 30 min, or with 5 µM BIS for 5 min, prior to stimulation with 1 U/ml thrombin (TH). Then, samples were incubated with anti-CD62P-FITC + anti-CD41-APC antibodies. Bar graphs represent the mean ± SEM of the percentage of P-selectin-FITC-labeled platelets normalized against TH-stimulated control platelets. & ≤ 0.05, &&& ≤ 0.001 *versus* their corresponding wild-type; ^†^p ≤ 0.05, ^††^p ≤ 0.01, ^†††^p ≤ 0.001 *versus* TH-stimulated wild-type; *p ≤ 0.05, **p ≤ 0.01, ***p ≤ 0.001 *versus* TH-stimulated tgC3G (left) or C3G-KO (right). *MFI* Mean Fluorescence Intensity, *a.u.* arbitrary units

We previously described that the transgenic expression of C3G in platelets induces the retention of some angiogenic factors (e. g. VEGF, bFGF and TSP-1) through a mechanism independent of its GEF activity [7], suggesting that C3G could participate in α-granule secretion. Consistently, ablation of C3G in platelets results in increased secretion of proangiogenic factors [6]. In agreement with these results, we show here that the secretome of thrombin-stimulated C3G-KO platelets contained a higher net amount of protein than that of C3G-wt platelets (Fig. 1A). C3G ablation did not produce substantial changes in platelet size or structure, nor did it affect the number of α- or δ-granules (Fig. S2A-C). Therefore, the observed increase in platelet secretion points to a faulty secretory mechanism. Supporting this, we found in C3G-KO platelets altered expression of some of the components of the secretion machinery: lower expression of P-selectin, VAMP-7 and Munc18-b, and higher expression of VAMP-3 and Stx11 mRNAs (Fig. 1B and Table S1). A decrease in P-selectin and VAMP-7 was also detected at the protein level (Fig. 1C) and by immunofluorescence in resting platelets (Fig. 1D, E). These findings align with a specific role of C3G in platelet secretion. Unexpectedly, we observed a positive correlation between C3G and RasGRP2 expression in MKs, potentially explaining why RasGRP2 is unable to compensate for the C3G defect (Fig. S1D).

C3G-KO platelets exhibit reduced levels of Rap1-GTP, integrin αIIbβ3 activation, platelet aggregation and P-selectin exposure on the surface in response to various agonists [4] (Fig. S1E–H). Lower levels of P-selectin were also detected in the membrane of thrombin-activated C3G-KO platelets by immunofluorescence, with only 50% of platelets exhibiting peripheral distribution *versus* 70% of C3G-wt platelets (Fig. 1D, F). In fact, P-selectin was retained in the cytoplasm of these platelets after stimulation with 0.2 U/ml thrombin (Fig. 1G). In addition, C3G deletion altered the distribution of VAMP-7, but not VAMP-8, α-granules in resting platelets. Indeed, we observed two different distribution patterns of VAMP-7: a normal central distribution (C3G-KO-1) and a peripheral/membrane distribution (C3G-KO-2) (Fig. S2D). Furthermore, we found an increase in the MFI of P-selectin and VAMP-7 in C3G-KO platelets after thrombin stimulation (Fig. 1D, E), which was not due to an increase in protein levels (Fig. 1H). Therefore, the increased MFI could be due to granule fusion.

In contrast to the role of C3G in regulating α-granule exocytosis, we did not observe any effect of C3G deletion in lysosome (Fig. S2E, F) or δ-granule secretion (Fig. S2G, H). Similarly, neither overexpression of C3G or C3GΔCat mutant, nor C3G ablation had any effect on platelet endocytosis (Fig. S2I).

Overall, these results indicate that C3G would play a role in platelet α-granule exocytosis, a process regulated by PKC [20]. Specifically, PKCδ activates thrombin-dependent degranulation [41]. On the other hand, C3G participates in the thrombin-PKC pathway leading to Rap1 activation in platelets, and is phosphorylated in a PKC-dependent manner [3, 4]. We observed that PKCδ inhibition (with Vtx27, an inhibitor of PKCδ and PKCθ isoforms), but not PKCθ or classical PKC inhibitors, abrogated the increased P-selectin exposure induced by C3G transgene expression, with a minor effect on C3G-KO platelets, suggesting that PKCδ is involved in the mechanism by which C3G regulates α-granule exocytosis (Fig. 1I). Consistently, PKCδ was also involved in the activation of integrin αIIbβ3 by C3G (Fig. S2J).

### C3G modulates the formation of Arp2/3-VARP-VAMP-7 and trans-SNARE complexes and participates in vesicle docking via Rap1-RalA

**Fig. 2:**
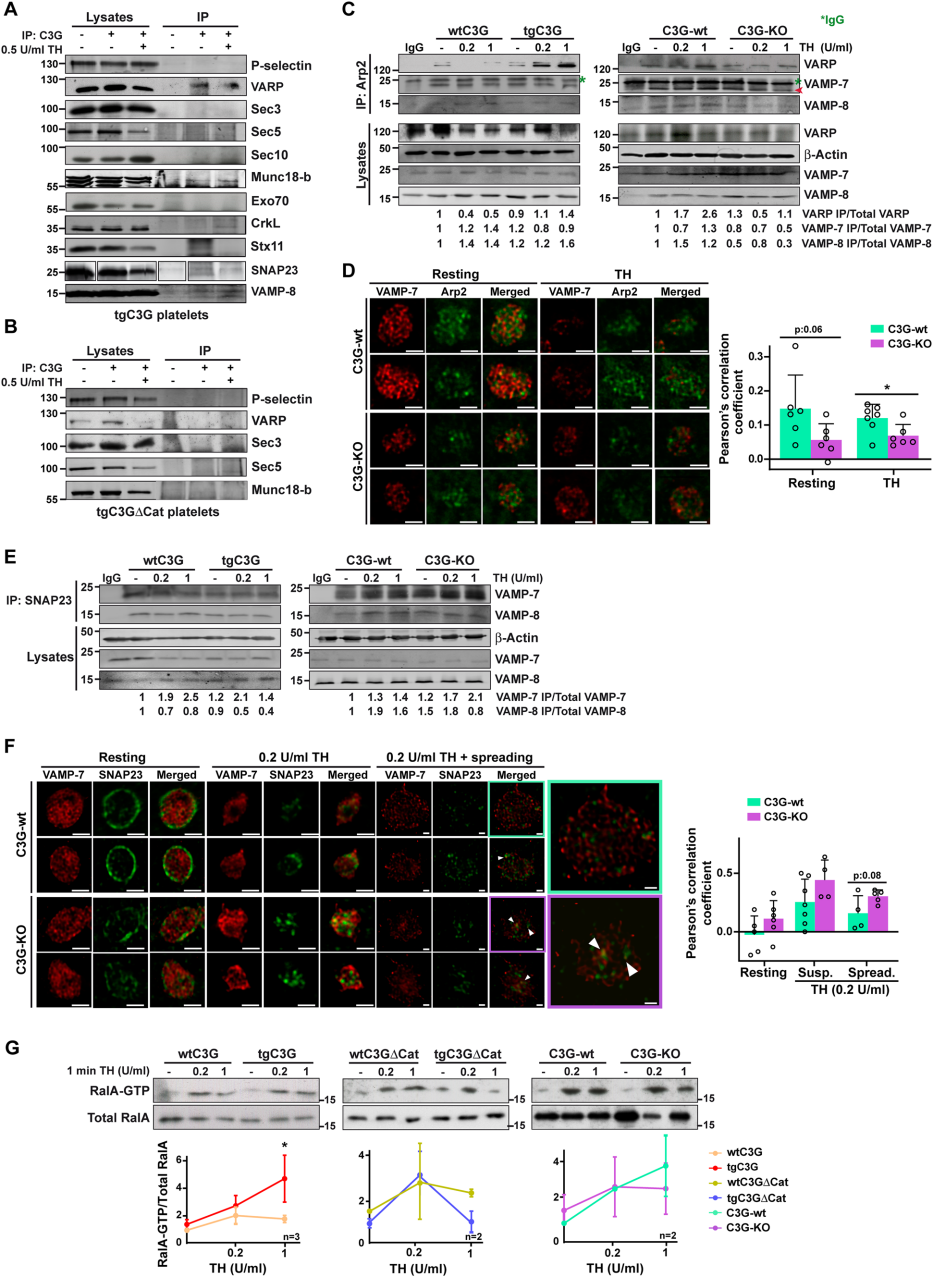
C3G interacts with proteins involved in vesicle trafficking and regulates Ral1 activation and the trans-SNARE and Arp-VARP-VAMP-7 complexes. **A** tgC3G or **B** tgC3GΔCat platelets, both at rest and after thrombin (TH) stimulation (0.5 U/ml for 1 min), were immunoprecipitated with anti-C3G G-4 antibodies and the levels of P-selectin, VARP, Sec3, Sec5, Munc18-b (tgC3G and tgC3GΔCat), Sec10, Exo70, CrkL, Stx11, SNAP23 and VAMP-8 (tgC3G) were detected by western blot. CrkL was used as a positive control. IP: immunoprecipitation; Stx11: Syntaxin 11. The SNAP23 panel in A is displayed as a composite panel. The raw data source file for this panel is illustrated in Fig. S5A. **C** Lysates from (left) wtC3G and tgC3G or (right) C3G-wt and C3G-KO platelets, both at rest and after thrombin (TH) stimulation (0.2 or 1 U/ml for 1 min) were immunoprecipitated with an anti-Arp2 antibody and the levels of VARP, VAMP-7 and VAMP-8 were detected by western blot. β-actin was used as loading control. IP: immunoprecipitation. Values are relative to corresponding total protein levels and were normalized against unstimulated wild-type platelets. Mouse IgG was used as a negative control. Green asterisks indicate non-specific bands (IgG). The red arrow points to VAMP-7 band. **D** Representative immunofluorescence confocal microscopy images of C3G-wt and C3G-KO platelets stimulated with 1 U/ml thrombin (TH) for 1 min and labeled with anti-VAMP-7 (H-55) + AF568 (red) and anti-Arp2 + AF647 (green). Scale bar: 2 µm. Bar graphs show the Pearson’s Correlation Coefficients (mean ± SD) of the intensity values of VAMP-7 and Arp2 under the indicated experimental conditions. **E** Lysates from (left) wtC3G and tgC3G platelets or (right) C3G-wt and C3G-KO platelets, both at rest and after thrombin (TH) stimulation (0.2 or 1 U/ml for 1 min) were immunoprecipitated with an anti-SNAP23 antibody and the levels of VAMP-7 and VAMP-8 detected by western blot. β-actin was used as loading control. IP: immunoprecipitation. Values are relative to levels in total lysate and are normalized to those of wild-type platelets. Mouse IgG was used as a negative control. **F** Representative immunofluorescence confocal microscopy images of C3G-wt and C3G-KO platelets stimulated with 0.2 U/ml thrombin (TH) for 1 min at rest or followed by 30 min of spreading, and labeled with anti-VAMP-7 (158.2) + AF647 (red) and anti-SNAP23 + AF568 (green). Scale bar: 2 µm. Enlarged images of a single platelet per genotype are depicted for a clearer visualization of colocalization. Arrowheads point to yellow pixels. Bar graphs show the Pearson’s Correlation Coefficients (mean ± SD) of VAMP-7 and SNAP23. **G** Pull-down assays to detect RalA activation after stimulation of tgC3G, tgC3GΔCat, C3G-KO platelets and their respective wild-types with 0.2 or 1 U/ml thrombin (TH) for 1 min. The levels of RalA-GTP were detected by immunoblotting with anti-RalA antibodies. Representative blots from 2–3 experiments are depicted. The line/scatter plots show the mean ± SD of RalA-GTP levels. Values are relative to total RalA levels. *p ≤ 0.05

We previously showed that C3G interacts with VAMP-7 protein, mainly with its SNARE domain, in resting and thrombin-stimulated platelets [7]. Consistent with that, and with the above results indicating a putative role for C3G in platelet secretion, we found that platelet C3G also interacted with secretory machinery proteins VARP and VAMP-8 and also weakly with SNAP23, Stx11, and Munc18-b (Fig. 2A). No interaction with VARP and Munc-18b was detected in tgC3GΔCat platelets, suggesting the involvement of the C3G GEF domain in the formation of these complexes (Fig. 2B). Likewise, no interaction of C3G with components of the exocyst complex, such as Sec3, Sec5, Sec10, or Exo70 was observed (Fig. 2A). Some of these interactions were confirmed in co-transfected HEK293T cells (Fig. S3A).

VARP forms a complex with Arp2 and VAMP-7 during platelet α-granule secretion, in which VARP prevents unscheduled actin polymerization, required for α-granule fusion and platelet spreading [12]. C3G overexpression in platelets favored the Arp2-VARP interaction, while a decrease in the interaction of Arp2 with VARP, VAMP-7 and VAMP-8 was found in C3G-KO platelets (Fig. 2C). In addition, significantly lower colocalization was also observed between VAMP-7 and Arp2 in resting and thrombin-stimulated C3G-KO platelets (Fig. 2D). These results point to a negative regulatory role of C3G in platelet degranulation.

The interaction with SNARE proteins suggests that C3G could participate in the formation of the trans-SNARE complex. Indeed, a stronger interaction between VAMP-7 and SNAP23 was found in thrombin-stimulated C3G-KO platelets, both in suspension and spreading conditions (Fig. 2E, F), whereas C3G overexpression resulted in decreased SNAP23/VAMP-7 complex formation (Fig. 2E). Similarly, interaction between VAMP-7 and Stx11 increased in spread C3G-KO platelets (Fig. S3B). Consistently, the absence of SNAP23 increased C3G interaction with VAMP-7 and Stx11, with no effect on the C3G-VARP interaction (Fig. S3C–F). These results indicate that C3G would compete with SNAP23 for binding to VAMP-7 and Stx11, and explain its lower interaction with SNAP23 and Stx11 in tgC3G platelets after stimulation (Fig. 2A). Therefore, C3G would act as a negative regulator of trans-SNARE complex formation.

Hence, our results suggest that C3G could act as a brake on platelet α-granule secretion by reinforcing the VARP-VAMP-7-Arp2 complex, thus preventing the SNAP23-VAMP-7 interaction and, consequently, the trans-SNARE complex formation. This would explain the increased secretion in C3G-KO platelets after thrombin stimulation (Fig. 1A).

RalA/B GTPases, whose activity is regulated by Rap1, are involved in targeting vesicles to the PM during platelet exocytosis [14]. Thus, we next studied whether C3G actions in exocytosis could be mediated by RalA/B activity. Figure 2G shows that overexpression of C3G significantly increased RalA-GTP levels after stimulation with 1 U/ml thrombin, whereas overexpression of the C3GΔCat mutant or C3G deletion showed the opposite tendency. This result indicates that C3G participates in Rap1-mediated RalA activation in platelets. Therefore, the defects in exocytosis observed in tgC3G and C3G-KO platelets could be explained by defects in docking/tethering (controlled by RalA activation) and also alterations in downstream steps, such as vesicle priming and fusion, regulated by the formation of the trans-SNARE complex.

### C3G promotes platelet spreading in a PKC- and substrate-dependent manner

**Fig. 3:**
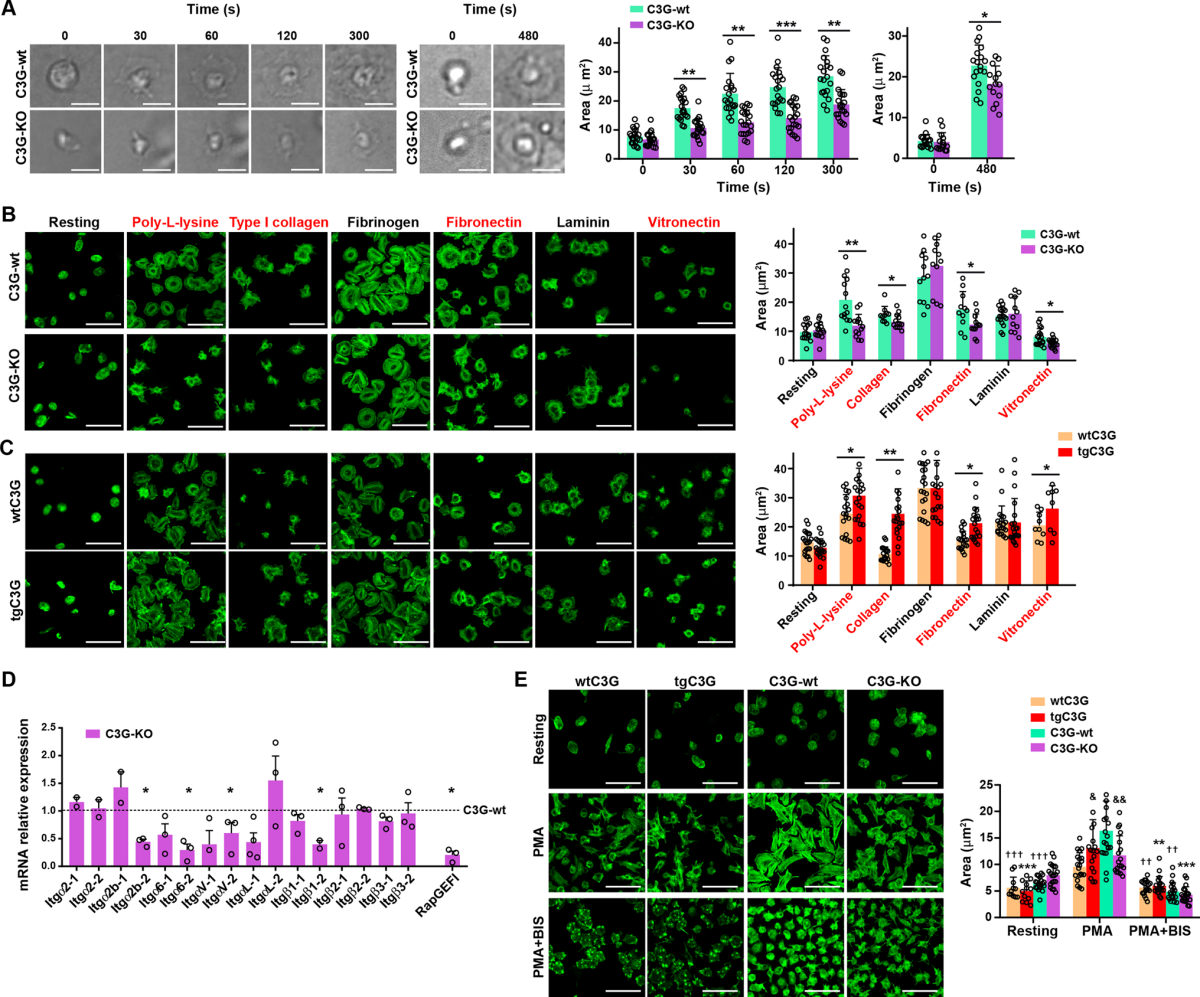
C3G promotes platelet spreading on poly-L-lysine, collagen, fibronectin and vitronectin through PKC.** A** Representative images of C3G-wt and C3G-KO platelets spread on ibiTreat plates at different times (0, 30, 60, 120, 300 and 480 s). Bar graphs represent the mean ± SD of platelet area, measured with ImageJ software. Scale bar: 5 µm. **B, C** Representative images of **B** C3G-wt and C3G-KO platelets or **C** tgC3G and wtC3G platelets resting or spread on poly-L-lysine, type I collagen, fibrinogen, fibronectin, laminin and vitronectin for 30 min after 0.2 U/ml thrombin stimulation. Platelets were stained with phalloidin-iFluor 488 to visualize actin cytoskeleton. Scale bar: 5 µm. Bar graphs represent the mean ± SD of platelet area (µm^2^). Substrates in which there are differences between genotypes are highlighted in red. **D** RT-qPCR analysis of the indicated integrin subunits in platelets from C3G-KO and C3G-wt mice using β-actin as housekeeping gene. Values were normalized to those of C3G-wt platelets. The expression of *RapGEF1* (C3G) was used as control. *p ≤ 0.05, **p ≤ 0.01, ***p ≤ 0.001. **E** Representative images of wtC3G, tgC3G, C3G-wt and C3G-KO platelets spread on poly-L-lysine for 30 min after pre-treatment or not with 5 µM BIS for 5 min, followed by 5 min stimulation with 2 µM PMA. Platelets were stained with phalloidin-iFluor 488 to visualize actin cytoskeleton. Scale bar: 5 µm. Bar graphs represent the mean ± SD of platelet area (µm^2^). & ≤ 0.05, && ≤ 0.01, *versus* their corresponding wild-types; ^††^p ≤ 0.01, ^†††^p ≤ 0.001 *versus* PMA-stimulated wild-type platelets; **p ≤ 0.01, ***p ≤ 0.001 *versus* PMA-stimulated tgC3G or C3G-KO platelets

One of the consequences of platelet degranulation is platelet spreading, which is dependent on VAMP-7 α-granules [12, 16]. We previously showed that transgenic expression of C3G increased platelet spreading in a Rap1-independent manner [7]. Consistently, C3G-KO platelets showed a marked delay in spreading on ibiTreat plates, as measured by decreased platelet area (Fig. 3A and Videos 1 and 2). To gain insight into this process, we evaluated whether the role of C3G in platelet spreading was substrate-dependent. In line with the above results, deletion of C3G in platelets resulted in defective spreading of thrombin-stimulated (0.2 U/ml) platelets on poly-l-lysine, type I collagen, fibronectin and vitronectin (Fig. 3B). In concordance, and in agreement with previous results [7], tgC3G platelets showed the opposite behavior on the same substrates (Fig. 3C). In contrast, C3G expression did not affect spreading on fibrinogen or laminin (Fig. 3B, C). These results indicate that the role of C3G in spreading depends on the substrate. In addition, tgC3GΔCat platelets exhibited increased spreading on poly-l-lysine and type I collagen, but decreased spreading on vitronectin, with no defects on fibronectin (Fig. S4A). This intermediate phenotype between that of tgC3G and C3G-KO platelets indicates that C3G would regulate platelet spreading through GEF-dependent and -independent mechanisms. Similar results were obtained with high dose thrombin (1 U/ml, data not shown).

The aforementioned results suggest that C3G could regulate the expression, or surface exposure, of integrins involved in the interaction of platelets with type I collagen, fibronectin or vitronectin. Thus, we analyzed in C3G-KO and control platelets the expression of subunits of the main platelet integrins, such as α2 (*ITGA2*), αIIb (*ITGA2B*), α6 (*ITGA6*), αV (*ITGAV*), αL (*ITGAL*), β1 (*ITGB1*), β2 (*ITGB2*) and β3 (*ITGB3*) by RT-qPCR [3, 4] using two different set of primers for each integrin (Table S1). We found significantly lower expression of the integrin subunits α6, αV and β1 in C3G-KO platelets than in C3G-wt platelets (Fig. 3D), which could explain the lower interaction of C3G-KO platelets on fibronectin, type I collagen and vitronectin [42]. However, we did not find alterations in the surface expression of GPVI (collagen receptor), integrins αIIb (ITGA2B or CD41) and β3 (ITGB3 or CD61), which bind to fibrinogen, fibronectin and vitronectin, and glycoprotein Ib alpha (GP1BA or CD42b), which binds to collagen, in tgC3G, tgC3GΔCat and C3G-KO platelets (Fig. S4B). This result suggests that C3G might be regulating the outside-in signaling triggered by collagen, fibronectin or laminin, probably affecting actin polymerization.

We next analyzed whether this C3G function was also dependent on PKC, given its involvement in platelet spreading [20]. As shown in Fig. 3E, the pan-PKC inhibitor bisindolylmaleimide (BIS) abrogated the differences in spreading on poly-l-lysine between the different genotypes after stimulation with PMA, indicating the involvement of PKC in the role of C3G in platelet spreading.

### C3G regulates actin polymerization during lamellipodia formation via Rac1, but independently of Rap1

**Fig. 4:**
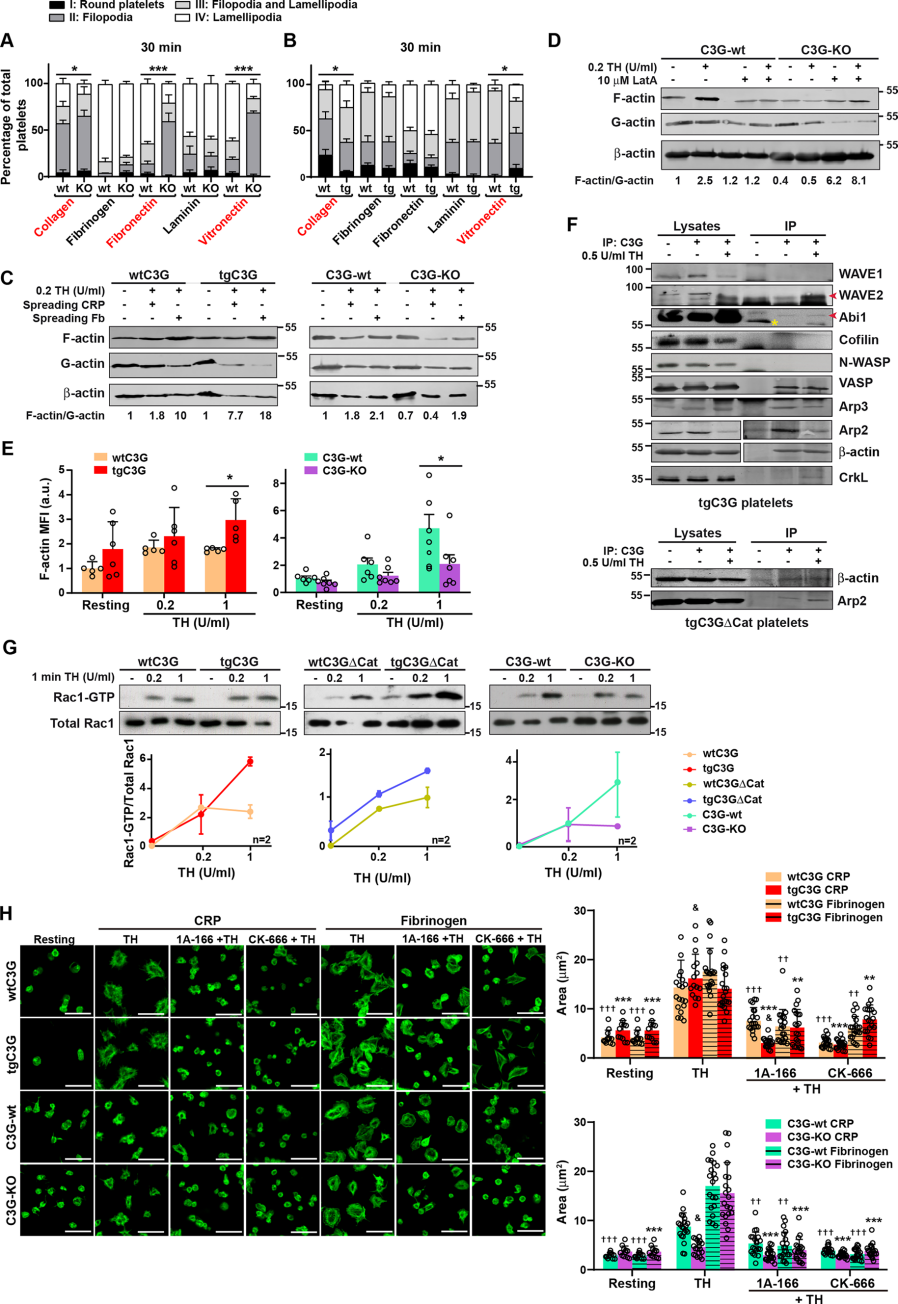
C3G interacts with proteins involved in actin remodeling and regulates lamellipodia formation through Rac1 in a Rap1-independent manner. **A, B** Quantification of the different spreading phases in fixed platelets from **A** C3G-wt and C3G-KO or **B** wtC3G and tgC3G mice, after 30 min spreading on type I collagen, fibrinogen, fibronectin, laminin or vitronectin coated coverslips, induced by 0.2 U/ml thrombin for 1 min. Bar graphs represent the mean ± SEM of the percentage of platelets in the different spreading phases: I: round platelets; II: platelets with filopodia; III: intermediate state with both filopodia and lamellipodia; IV: spread platelets. wt: wild-type; tg: transgenic; KO: knockout. Asterisks refer to quantification of platelets in phase IV. Substrates showing differences between genotypes are highlighted in red. **C, D** Platelets from wtC3G and tgC3G or C3G-wt and C3G-KO mice were stimulated with 0.2 U/ml thrombin (TH) for 1 min and allowed to spread on CRP-XL (CRP) or fibrinogen (Fb) for 30 min before lysis (**C**), or C3G-wt and C3G-KO platelets in suspension were pre-treated with 10 µM latrunculin (LatA) for 45 min and stimulated with 0.2 U/ml thrombin (TH) for 1 min before lysis (**D**). The supernatant containing the soluble G-actin and the urea-solubilized triton X-100-insoluble pellet containing F-actin were subjected to SDS-PAGE and the levels of F-actin and G-actin were detected with anti-β-actin antibodies. Levels of β-actin from total lysates are also depicted. The numbers indicate the ratio F/G actin normalized to wild-type, non-treated platelets. Fb: Fibrinogen. **E** Washed platelets in suspension from (left) wtC3G and tgC3G or (right) C3G-wt and C3G-KO mice were stimulated with 0.2 or 1 U/ml thrombin (TH) for 1 min before fixing with 2% PFA. After permeabilization with 0.2% Triton X-100, platelets were stained with phalloidin-iFluor 488 and analyzed by flow cytometry. Bar graphs represent the mean ± SD of phalloidin-iFluor 488 MFI, normalized against resting control platelets. **F** Lysates from (upper) tgC3G and (lower) tgC3GΔCat platelets, resting or stimulated with thrombin (TH, 0.5 U/ml for 1 min) were immunoprecipitated with anti-C3G antibodies (anti-C3G G-4 in the case of WAVE1, Cofilin, Arp3; anti-C3G C-19 in the case of Abi1, β-Actin, WAVE2, Arp2 and CrkL) and the levels of WAVE1, WAVE2, Abi1, Cofilin, N-WASP, β-actin, VASP, Arp2, Arp3 and CrkL were detected by western blot. CrkL was used as a positive control. Red arrows indicate WAVE2 and Abi1 bands. The yellow asterisk indicates a non-specific band. IP: immunoprecipitation. The Arp2 and β-actin western blots and IPs are shown as composite panels. The raw data source file for these panels is depicted in Fig. S5B. **G** Pull-down assay to detect Rac1 activation after stimulation of tgC3G, tgC3GΔCat, C3G-KO platelets and their respective wild-types with 0.2 or 1 U/ml thrombin (TH) for 1 min. The levels of Rac1-GTP were determined using anti-Rac1 antibodies. Representative blots from 2 experiments are depicted. The line/scatter plots show the mean ± SD of Rac1-GTP levels. Values are relative to total Rac1 levels. *p ≤ 0.05, ***p ≤ 0.001. **H** Representative images of wtC3G, tgC3G, C3G-wt and C3G-KO platelets spread on CRP-XL (CRP) or fibrinogen for 30 min after pre-treatment with 50 µM 1A-166 or 50 µM CK-166 for 30 min, prior stimulation with 0.2 U/ml thrombin (TH) for 1 min. Platelet were stained with phalloidin-iFluor 488 to visualize actin cytoskeleton. Scale bar: 5 µm. Bar graphs represent the mean ± SD of platelet area (µm^2^) of (upper) wtC3G and tgC3G or (lower) C3G-wt and C3G-KO platelets. & ≤ 0.05 *versus* its wild-type. ^††^p ≤ 0.01, ^†††^p ≤ 0.001 *versus* TH-stimulated wild-type spread platelets; **p ≤ 0.01, ***p ≤ 0.001 *versus* TH-stimulated tgC3G or C3G-KO spread platelets. *MFI* Mean Fluorescence Intensity, *a.u.* arbitrary units

Platelets spread by forming filopodia and lamellipodia, which involves profound changes in the actin cytoskeleton [43]. The in vivo spreading experiment in Fig. 3A, suggested defective lamellipodia formation in C3G-KO platelets. Based on that, we studied whether the abnormal spreading found in C3G-KO and tgC3G platelets were due to defective formation of these actin structures. To do so, we performed a time-lapse spreading assay, on different substrates, on thrombin-stimulated platelets, analyzing the formation of filopodia and lamellipodia after 5, 15, and 30 min of spreading. C3G-KO platelets were able to form filopodia on all substrates, but showed delayed lamellipodia formation on type I collagen, fibronectin, and vitronectin (Fig. 4A and Fig. S4C), the same substrates on which defective spreading was observed. Consistently, C3G overexpression triggered faster lamellipodia formation on type I collagen and vitronectin, although with smaller differences (Fig. 4B and S4D).

The above results suggest a role for C3G in actin remodeling during the outside-in signaling. Therefore, we monitored actin fiber formation in tgC3G, C3G-KO platelets and their wild-types spread on CRP-XL and fibrinogen for 30 min, by analyzing the F-actin/G-actin ratio by western blot. As shown in Fig. 4C, C3G overexpression induced a slight increase in actin polymerization after spreading on CRP-XL, but not on fibrinogen. Consistently, and most evidently, C3G-KO platelets showed a decrease in F-actin levels after spreading on CRP-XL (Fig. 4C) or in suspension (Fig. 4D, E), but not on fibrinogen (Fig. 4C). All these results support a regulatory role for C3G in platelet cytoskeleton remodeling during spreading on type I collagen, but not on fibrinogen, consistent with the results in Fig. 3B–D. All these results suggest the involvement of C3G in outside-in signaling triggered by type I collagen, fibronectin and vitronectin, leading to actin polymerization.

To further characterize this role of C3G in spreading and since Src kinases regulate C3G phosphorylation [4] and platelet spreading [44], we analyzed spreading in the presence of the Src inhibitor PP2. PP2 abolished the increase in platelet area in tgC3G platelets, suggesting the involvement of Src in this C3G function (Fig. S4E). However, PP2 did not inhibit spreading in control platelets at the concentration and time used. Alternative pathways regulating platelet spreading in the absence of Src have been described [44]. Furthermore, we analyzed platelet spreading in tgC3G, C3G-KO and control platelets in the presence of reagents that interfere with actin polymerization, such as cytochalasin D (CytD) or latrunculin A (LatA). As shown in Fig. S4E, and as expected, CytD-and LatA-treated tgC3G and wild-type platelets were unable to spread. In contrast, C3G-KO platelets were affected by CytD, but not LatA treatment, which rather reverted the effect of C3G knockout. This suggests that C3G deletion would prevent the effect of LatA, which binds actin monomers and enhances F-actin depolymerization from both ends [45]. These data are reinforced by the observation that LatA reverted the decrease in F-actin/G-actin ratio induced by C3G deletion (Fig. 4D).

Given this new role of C3G in regulating actin cytoskeletal structures in platelets, we next examined whether C3G interacts with proteins involved in cytoskeletal remodeling. Immunoprecipitation assays showed that C3G interacted with WAVE2, Abi1, VASP, Arp2, and Arp3, but not with WAVE1, Cofilin, and N-WASP in resting and thrombin-stimulated platelets (Fig. 4F). CrkL and β-actin were monitored as positive controls of C3G interactions. Similar results were obtained in co-transfected HEK293T cells (Fig. S4F). These results suggest that C3G could regulate Rac1 activity in platelets. Indeed, we found higher levels of Rac1-GTP in thrombin-stimulated tgC3G platelets, compared to control platelets, while C3G-KO platelets showed the opposite trend (Fig. 4G). Surprisingly, overexpression of the C3GΔCat mutant also promoted Rac1 activation, suggesting that C3G would regulate Rac1 activity independently of Rap1 (Fig. 4G). No effects of C3G on RhoA or Cdc42 activation were detected (data not shown). To corroborate these findings, we monitored platelet spread on CRP-XL and fibrinogen in thrombin-stimulated tgC3G, C3G-KO platelets, and their controls in the presence of 1A-166 or CK-666, inhibitors of Rac1 and Arp2/3 complex, respectively. As expected, both inhibitors significantly reduced spreading on both substrates in all genotypes. However, tgC3G platelets spread on CRP-XL (but not on fibrinogen) were more sensitive to Rac1 and Arp2/3 inhibition than wtC3G platelets (Fig. 4H). This result supports the involvement of C3G in the Arp2-VARP-VAMP-7 complex (Fig. 2C), and further suggests that C3G would regulate Rac1 function during platelet spreading.

### C3G deletion in platelets promotes kiss-and-run and compound exocytosis

**Fig. 5:**
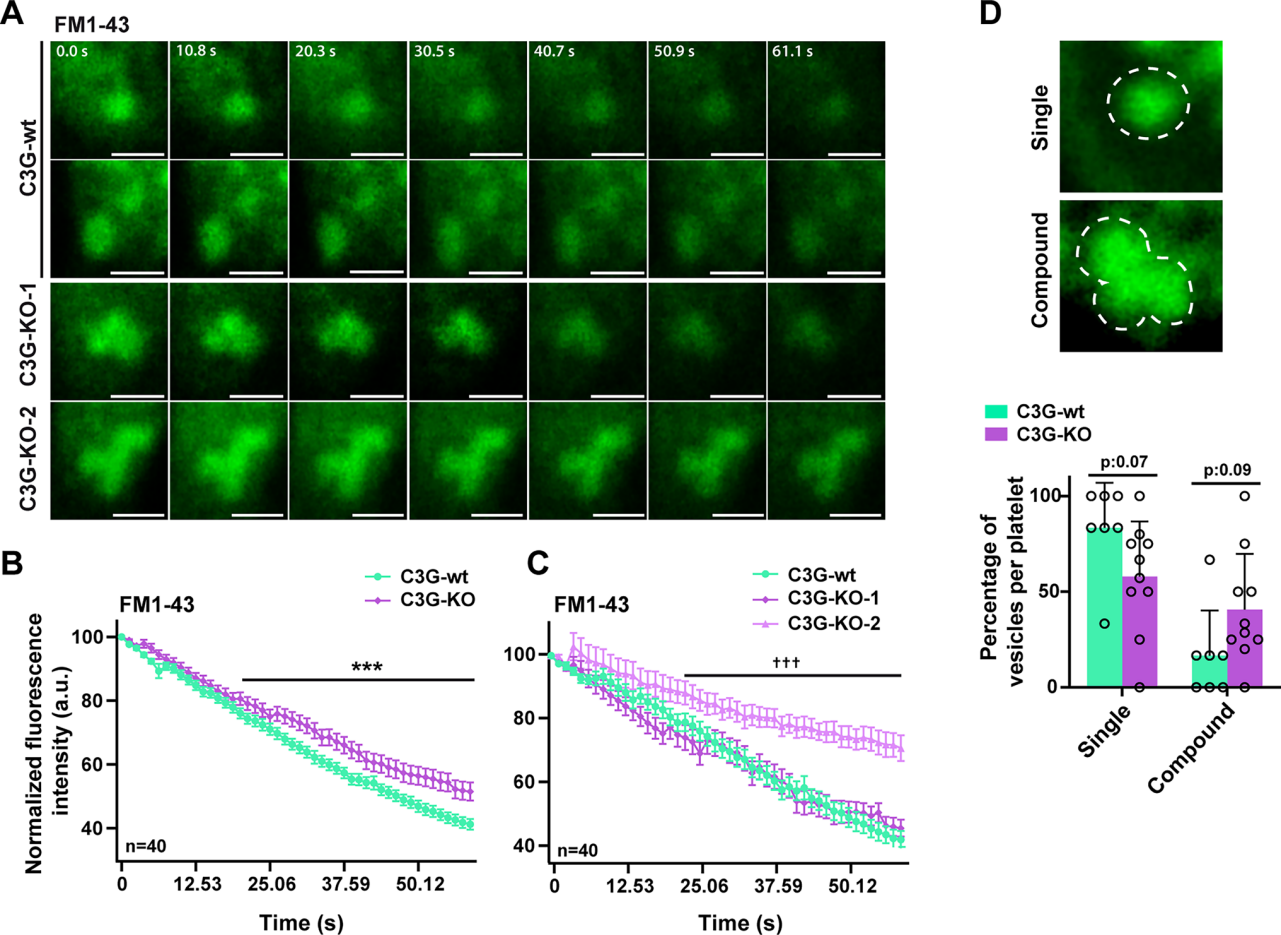
C3G-KO platelets show kiss-and-run and compound exocytosis. C3G-wt and C3G-KO platelets were allowed to spread on a µ-Slide 8-well plate (Ibidi) after staining with 10 µm FM1-43. **A** Representative images of granules, taken every 10 s for 1 min. **B** Scatter plot representing the quantification of MFI (mean ± SEM) of individual FM1-43-stained granules from all C3G-wt and C3G-KO platelets analyzed. **C** Same analysis as in **B** but C3G-KO platelets were separated into two categories depending on their phenotype: fuse and collapse (C3G-KO-1) or kiss-and-run (C3G-KO-2) exocytosis. ***p ≤ 0.001. ^†††^p ≤ 0.001 C3G-KO-2 *versus* C3G-wt. **D** Representative images showing single and compound granules. Bar graphs represent the mean ± SD of the percentage of single or compound vesicles in platelets of each genotype

So far, our results indicate that C3G would favor platelet spreading by promoting the Rac1-WAVE2-Arp2/3 pathway, while it would decrease platelet secretion by interfering with the trans-SNARE complex. The opposite was found in C3G-KO platelets, i.e., increased platelet secretion and decreased spreading. The C3G-KO platelet phenotype is consistent with a kiss-and-run exocytosis, similar to that seen in RalA/B double KO platelets [13]. To verify this, we monitored the spreading of C3G-KO and C3G-wt platelets on ibiTreat plates (Ibidi) after staining with FM1-43. While C3G-wt platelets showed decreased fluorescence over time, indicating a normal, fuse and collapse, exocytosis, two different exocytosis patterns were found in C3G-KO platelets: (i) fuse and collapse exocytosis (C3G-KO-1), with kinetics similar to that of C3G-wt platelets, (ii) kiss-and-run exocytosis (C3G-KO-2), in which FM1-43 signal is not lost, indicating no fusion of the granules with the PM (Fig. 5A-C). In addition, we observed a higher number of fused granules in C3G-KO platelets, compared to C3G-wt platelets, indicative of compound exocytosis (Fig. 5D), in agreement with results in Fig. 1D, E.

### C3G regulates granule exocytosis in PC12 cells, promoting protein release

**Fig. 6:**
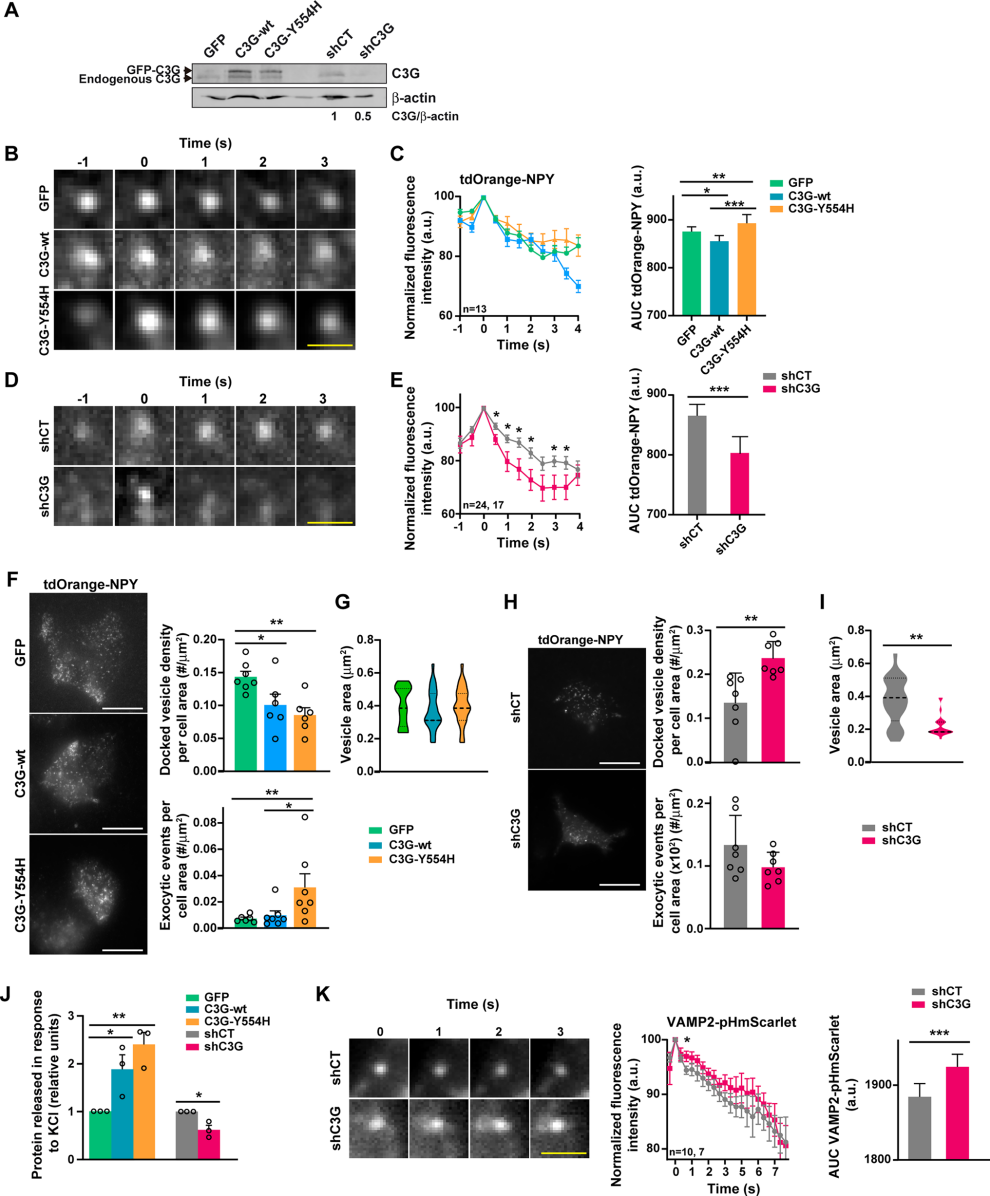
C3G regulates granule secretion kinetics in PC12 cells. PC12 clones expressing GFP, C3G-wt and C3G-Y554H were generated with lentiviral plasmid pLenti-C-mEGFP-IRES-BSD. The shCT and shC3G PC12 clones were generated with plasmid pLVTHM. **A** Representative western blot showing C3G expression in PC12 clones, detected with the anti-C3G F-5 antibody. β-actin was used as loading control. The C3G/β-actin ratio is shown to illustrate C3G silencing. **B, D** Representative sequential images of a single tdOrange-NPY vesicle observed after 56 mM KCl stimulation of **B** GFP-expressing cells, cells expressing C3G-wt and cells expressing the active mutant C3G-Y554H, or **D** control cells (shCT) and C3G silencing cells (shC3G), acquired at 300-ms intervals, through a TIRF microscope. Scale bar: 1 µm. **C, E** Line/scatter plots (left) represent the mean ± SEM of the time course of fluorescence intensity changes measured in tdOrange-NPY vesicles. The MFI at the time of fusion (maximum intensity) was set to 100%. (B, C: n = 13 vesicles in each experiment; D, E: n = 24 and n = 17 vesicles in shCT and shC3G experiments, respectively). Bar graphs (right) represent the mean ± SD of AUC (area under the curve) of the fluorescence intensity changes measured in the center of tdOrange-NPY vesicles. **F, H** Representative images of tdOrange-NPY in **F** GFP, C3G-wt and C3G-Y554H expressing PC12 cells or **H** shCT and shC3G expressing PC12 cells, acquired through a TIRF microscope. Scale bar: 20 µm. Bar graphs represent (upper) the mean ± SD of the number of docked vesicles per cell, relativized to cell area and (lower) the number of exocytic events per cell area, after KCl stimulation monitored for 1 min. **G, I** Violin plots represent the median and whiskers of the 25th and 75th percentiles of vesicle area (n = 7). **J** Bar graphs represent the mean ± SEM of the amount of protein released after 56 mM KCl stimulation at 37 °C for 1 h (values normalized to those of the GFP clone). **K** Representative sequential images of a single VAMP-2-pHmScarlet vesicle observed after 56 mM KCl stimulation of control (shCT) and C3G-silenced (shC3G) PC12 cells, acquired at 300-ms intervals, through a TIRF microscope. Scale bar: 1 μm. Line/scatter plots (left) represent the mean ± SEM of the time course of MFI changes measured in VAMP-2-pHmScarlet vesicles. The MFI at the time of fusion (maximum intensity) was set to 100%. n = 10 and n = 7 vesicles in shCT and shC3G experiments, respectively. Bar graphs (right) represent the mean ± SD of AUC (area under the curve) of MFI changes measured in the center of VAMP-2-pHmScarlet vesicles. *p ≤ 0.05, **p ≤ 0.01, ***p ≤ 0.001. *NPY* neuropeptide Y, *MFI* Mean Fluorescence Intensity, *a.u.* arbitrary units

To further demonstrate the involvement of C3G in granule secretion, we investigated whether C3G modulates the kinetics of dense-core vesicle exocytosis in NPY-td-Orange- expressing PC12 clones stably transfected with wild-type C3G (C3G-wt), a hyperactive C3G mutant (C3G-Y554H) [34], or a shC3G construct (pLVTHM-C3Gi) to silence C3G expression [5] (Fig. 6A). We used TIRF Microscopy to analyze the dynamics of vesicle-PM fusion events, by monitoring the kinetics of the tdOrange signal after KCl stimulation, a stimulus of granule exocytosis in these cells. Overexpression of wild type C3G resulted in a slightly lower AUC of tdOrange fluorescence, indicating faster secretion kinetics. In contrast, overexpression of C3G-Y554H mutant significantly slowed the kinetics of tdOrange-NPY release, manifested by increased AUC (Fig. 6B, C and Videos 2–5). On the other hand, C3G-silencing significantly increased the rate of tdOrange-NPY release (Fig. 6D, E and Videos 6 and 7). Next, we analyzed the number of secretion events that occurred in our C3G mutant PC12 cells. As shown in Fig. 6F, overexpression of the C3G-Y554H mutant decreased the number of docked vesicles, but increased the number of exocytic events, indicating that C3G would interfere with docking but facilitate priming and fusion. Overexpression of wild type C3G also induced a lower number of docked vesicles but did not result in changes in the number of exocytic events. Neither C3G-wt nor C3G-Y554H modified granule area (Fig. 6G). In contrast, shC3G cells displayed a significantly higher number of docked vesicles, while presenting a lower number of exocytic events after KCl stimulation (Fig. 6H). Furthermore, shC3G cells had smaller granules, compared to shCT cells (Fig. 6I).

Next, we quantified protein secretion in C3G PC12 mutants. Overexpression of C3G-wt and C3G-Y554H mutant induced increased protein secretion, compared to the GFP control. Consistently, shC3G cells secreted a lower amount of protein that shCT cells (Fig. 6J).

Finally, we investigated pore formation and expansion, using the VAMP2-pHmScarlet plasmid, which monitors the connection of the vesicle to the extracellular space by measuring changes in pH [36]. C3G-silenced PC12 cells displayed a higher MFI of VAMP2-pHmScarlet, suggesting that C3G absence promotes greater pore expansion and faster incorporation of the vesicle membrane into the PM (Fig. 6K and Videos 8 and 9), in agreement with the faster exocytosis observed (Fig. 6D, E).

Overall, these data indicate that C3G would regulate granule secretion in PC12 cells after KCl stimulation. C3G hyperactivation induced slower secretion but a greater number of exocytic events and increased protein release, whereas C3G silencing triggered the opposite phenotype, i.e., faster but less frequent secretion. In addition, C3G silencing promotes faster vesicle membrane incorporation, due to increased pore opening and expansion, promoting a fuse and collapse exocytic mode. Although the actions of C3G on PC12 exocytosis are opposite to those on platelets, these data confirm the role of C3G in regulating granule secretion and suggest a more general role for C3G in this process.

### C3G ablation impairs thrombin production and clot retraction, but favors the release of coagulation factors

**Fig. 7:**
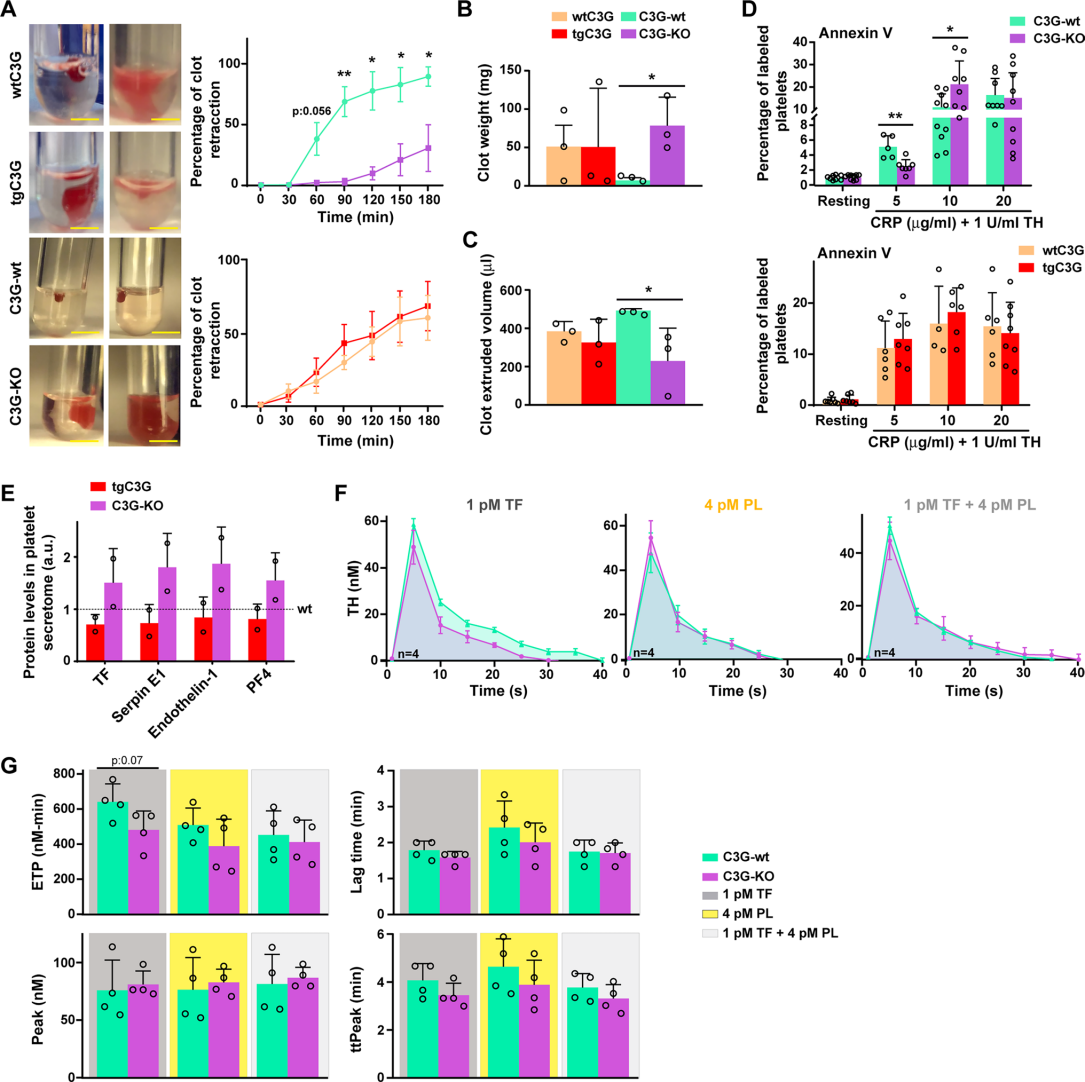
C3G regulates secondary hemostasis and clot retraction.** A** Representative images and quantification of the ability of clot retraction of PRP from wtC3G and tgC3G mice or C3G-wt and C3G-KO mice. Clot formation was initiated by adding 1 U/ml thrombin and photographed each 30 min for 3 h. The images (2 per genotype) correspond to the end point of the experiment. Scale bar: 5 mm. Line/scatter plots represent the mean ± SEM. **B** Clot weight and **C** clot extruded volume was monitored at the end point of the experiment. Bar graphs represent the mean ± SEM. **D** Washed platelets from wtC3G and tgC3G mice or C3G-wt and C3G-KO mice were stimulated with 1 U/ml thrombin (TH) in combination with 5, 10 or 20 µg/ml CRP-XL (CRP), and labeled with anti-CD41-FITC antibodies and Annexin V-APC for 15 min. Samples were diluted and analyzed by flow cytometry. Bar graphs represent the mean ± SD of the percentage of double labeled events. **E** Secretome from thrombin-stimulated tgC3G, C3G-KO platelets, and their corresponding wild-types were analyzed using the commercial Mouse Angiogenesis Array Kit. Bar graphs represent the quantification (mean ± SD) of TF, Serpin E1, Endothelin-1, and PF4 levels. Data were normalized against their corresponding control (wild-type), which was given a value of 1 in each independent experiment. **F** Thrombin (TH) generation detected by Calibrated Automated Thrombinography (CAT) of PRP from C3G-wt and C3G-KO mice in response to (left) 1 pM TF, (middle) 4 pM PL and (right) the combination of both. **G** Bar graphs represent the mean ± SD of the endogenous thrombin potential or ETP, lag time, peak thrombin generation or Peak, and time to peak or ttPeak. *PF4* platelet factor 4, *TF* tissue factor, *PL*:phospholipids. *p ≤ 0.05, **p ≤ 0.01. *a.u.* arbitrary units

Rap1 regulates platelet spreading and clot retraction via Rac1 [25]. Retraction of fibrin clots involves activation of platelet integrins and their connection to actin cytoskeleton [46]. Given the role of C3G in integrin activation [3, 4], actin cytoskeleton remodeling and Rac1 activation, we examined the participation of C3G in clot retraction. C3G-KO platelets were defective in thrombin-induced clot retraction (Fig. 7A), showing greater clot weight (Fig. 7B) and lower extruded volume (Fig. 7C), compared to C3G-wt platelets. In contrast, overexpression of C3G had no effect in this process (Fig. 7A-C). This suggests that a minimal level of C3G is required for platelet clot retraction.

We have previously demonstrated a role for C3G in primary hemostasis [3, 4]. The above results suggest that C3G might also participate in secondary hemostasis, which involves the formation of fibrin by thrombin to stabilize the platelet plug. Platelets facilitate thrombin generation by exposing PS [47]. Therefore, we first determined whether C3G regulates PS exposure on platelet surface upon stimulation with a combination of thrombin and increasing concentrations of CRP-XL. C3G-KO platelets showed lower surface PS exposure at 5 µg/ml CRP-XL, compared to C3G-wt platelets (Fig. 7D). However, they showed increased PS translocation after stimulation with 10 µg/ml CRP-XL (Fig. 7D), suggesting a compensatory mechanism to counteract the delay of these platelets in PS exposure. Overexpression of C3G in platelets had no effect in PS externalization upon thrombin-CRP-XL mixture stimulation (Fig. 7D). We next examined whether C3G modulates the secretion of factors of the coagulation cascade. As shown in Fig. 7E, C3G ablation triggered a marked increase in the secretion of tissue factor (TF), Serpin E1, Endothelin-1, and PF4, upon thrombin stimulation, whereas C3G overexpression caused their retention, in line with previous results on the secretion of angiogenic factors [6, 7]. Finally, we analyzed whether alterations in PS exposure or coagulation factor secretion in C3G-KO platelets could affect thrombin production. Using CAT technique, we examined thrombin generation after stimulation with TF, phospholipids or a combination of both. C3G-KO platelets displayed lower thrombin generation in response to TF. Specifically, C3G-KO platelets showed a nearly significant decrease in ETP (AUC) in response to TF (Fig. 7F, G), and a decreased tendency in lag time and time to peak (ttPeak). Therefore, the increased amount of TF secreted by C3G-KO platelets cannot compensate for defective PS exposure, leading to decreased thrombin generation, which could explain the reduced clot retraction observed.

Taken together, our results indicate that C3G regulates outside-in signaling, leading to platelet spreading and clot retraction through regulation of actin cytoskeleton dynamics.

## Discussion

In the present work, we provide new insights into the involvement of C3G in platelet functions. These include data on its role in granule secretion and spreading, as well as in secondary hemostasis and clot retraction.

We have previously described that C3G modulates the secretion of some angiogenic factors from platelets. Specifically, C3G overexpression causes the retention of pro-angiogenic factors VEGF and bFGF, as well as the anti-angiogenic factor TSP-1 [7], while C3G ablation increases the release of VEGF and SDF-1 after thrombin stimulation [6, 7]. In concordance, we found that C3G-KO platelets released a higher overall amount of protein compared to C3G-wt platelets. All of these data point to a role of C3G in the secretion of platelet granules. The defective granule secretion observed in our C3G mutant platelets was not due to changes in granule number, and was only associated with α-granules, but not with δ-granules or lysosomes. Interestingly, despite their increased protein secretion from α-granules, C3G-KO platelets display defective P-selectin exposure on the platelet surface [4], suggesting a kiss-and-run phenotype.

Furthermore, in C3G-KO platelets, the levels of P-selectin and VAMP-7 (MFI signal) increased after activation, despite their lower basal levels, indicating that C3G absence in platelets would favor a compound exocytosis. This was demonstrated in FM1-43-stained C3G-KO platelets. In addition, VAMP-7 α-granules exhibited two different distribution patterns in C3G-KO platelets; (i) granules distributed throughout the platelet with an accumulation in the center and (ii) a more peripheral distribution, closer to the PM. Under resting conditions, α-granules are separated from each other by cytoplasmic actin filaments, which would act as a brake on granule secretion, while after activation they move to the platelet center for secretion through the open canalicular system [48]. Since the absence of C3G induced defects in actin filament formation, these defects could cause the peripheral distribution of VAMP-7 granules observed in C3G-KO platelets, favoring secretion through a secondary mechanism involving the direct fusion of the granules with the PM [48].

In platelets, C3G is activated in a PKC-dependent manner, both participating in a common pathway that activates Rap1 by thrombin, independently of Ca^2+^ [3, 4, 49, 50]. The fact that this pathway does not require Ca^2+^ suggests the participation of a novel PKC isoform. Indeed, we showed that the role of C3G in platelet activation, including platelet secretion, appears to be dependent on PKCδ.

Consistent with these defects in α-granule distribution and secretion, C3G-KO platelets showed altered levels of proteins involved in the secretion machinery. In particular, a decrease in P-selectin expression and its accumulation in the cytosol of C3G-KO platelets was observed after thrombin stimulation, in accordance with its lower presence on the platelet surface after stimulation. [4]. This indicates a defect in α-granule fusion with the PM, compatible with a kiss-and-run phenotype.

**Fig. 8:**
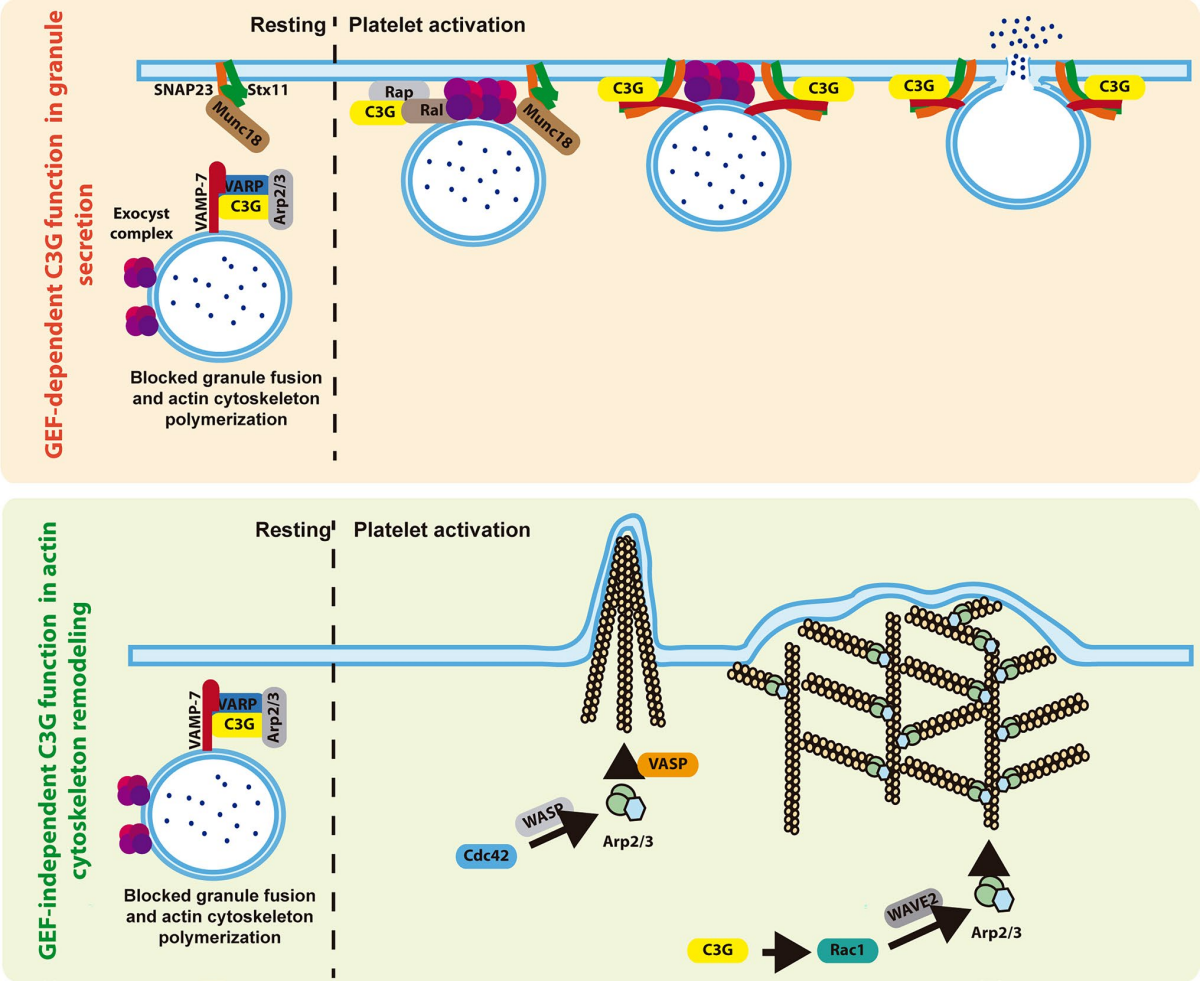
Schematic representation of the participation of C3G in α-granule secretion and platelet spreading. Under resting conditions (left of the dotted line), C3G would form a complex with VARP, VAMP-7 and Arp2/3, thus preventing uncontrolled secretion of α-granules (upper) or actin polymerization (lower). After stimulation (right of the dotted line), C3G would participate in vesicle tethering/docking and in the trans-SNARE complex formation, through Rap1-mediated Ral activation (upper). C3G would also participate in actin branching through the regulation of the Rac1/WAVE2/Arp2/3 pathway, independently of Rap1, thus promoting lamellipodia formation (lower)

In concordance with this role of C3G as a modulator of the secretory machinery, we previously demonstrated that C3G interacts (directly or through other proteins) with VAMP-7 [7]. Here, we show that C3G also formed complexes with VAMP-8 and VARP, and very likely with t-SNAREs, such as SNAP23 and Stx11, and with regulators, such as Munc18-b. These interactions were not detected in tgC3GΔCat platelets, supporting the involvement of Rap1b in the role of C3G in secretion. Hence, C3G could modulate the formation of the trans-SNARE complex, in which C3G and SNAP23 would compete for binding to VAMP-7 [7, 51]. Indeed, we observed a decrease in trans-SNARE complex formation in tgC3G platelets and an increase in C3G-KO platelets. In addition, our results suggest that C3G reinforces the Arp2/3-VARP-VAMP-7 complex, so C3G could act as a degranulation break [12]. This would explain the retention of α-granule factors in tgC3G platelets and the increased release in C3G-KO platelets (Fig. 8).

Consistent with the above comments and with other works [52], C3G promoted Ral activation through Rap1. Interestingly, RalA and RalB double knockout platelets exhibit a kiss-and-run phenotype, similar to that seen in C3G-KO platelets [13], further supporting a functional relationship between these proteins. In addition, RalA/B knockout platelets also exhibit reduced P-selectin exposure on the platelet surface, although they release cargo from α-granules normally [13].

All these results support the participation of C3G in α-granule secretion at two different levels. On the one hand, it would favor tethering/docking by promoting Ral GTPase activation. On the other hand, it would modulate the formation of the VARP-Arp2/3-VAMP-7 complex, probably sequestering VAMP-7, so that VAMP-7 would not be available to interact with SNAP23, thus restricting granule fusion and cargo release (Fig. 8).

The role of C3G in granule secretion is not limited to platelets, as it was also observed in the PC12 cell line. Overexpression of an active C3G mutant induced slower NPY secretion, consistent with a lower number of vesicles docked to the PM, whereas C3G absence triggered the opposite phenotype. However, C3G hyperactivation increased the number of exocytic events in PC12 cells, which overall resulted in higher levels of protein released. Therefore, in PC12 cells, C3G would promote granule priming/fusion, while impairing docking, opposite behavior to that observed in platelets. This contradictory phenotype could be explained by the different functionality of the VARP-VAMP-7 complex in platelets and neurons. In neurons, VARP positively regulates VAMP-7, inducing the formation of the trans-SNARE complex [53]. However, as mentioned, in platelets the VAMP-7-VARP interaction promotes the opposite effect [12].

Furthermore, C3G appears to play a role in pore formation/expansion. In kiss-and-run events, pore formation is transient [54]. Thus, a possible explanation for the defective spreading of C3G-KO platelets, despite increased trans-SNARE complex formation, could be a defect in pore expansion, resulting in a kiss-and-run phenotype.

We have previously shown that overexpression of C3G in platelets enhances platelet spreading on poly-L-lysine by a Rap1-independent mechanism [7]. In agreement, C3G-KO platelets displayed defective spreading due to their inability to transition from filopodia to lamellipodia, resulting in sustained filopodia. This phenotype was similar to that of PKCδ-KO platelets [21], further supporting the involvement of this novel PKC isoform in C3G actions on platelets. Indeed, C3G-mediated increase in spreading was abrogated by PKC inhibition. C3G has also been linked to the regulation of filopodia in HeLa cells [55]. C3G also required Src for platelet spreading, consistent with the role of this kinase in this process [56], and in C3G activation in platelets and other cell types [4, 18, 57].

In addition, C3G regulated spreading in a substrate-dependent manner: it promoted platelet spreading on poly-L-lysine, collagen/CRP-XL, fibronectin and vitronectin, but not on fibrinogen or laminin. Supporting this, C3G stimulated lamellipodia formation in the same substrates. This phenotype has been previously described in the literature. For instance, PKCθ regulates platelet spreading on fibrinogen but not on collagen/CRP-XL [58], and platelets knockout for Tspan18 (a regulator of thromboinflammation) show defective spreading on collagen but not on fibrinogen [59]. The decreased expression of integrins α6, αV, and β1 in C3G-KO platelets could partially explain this phenotype [42]. However, there were no differences in the levels of the main receptors on the platelet surface, suggesting that spreading on certain substrates would depend more on proper actin polymerization, triggered during outside-in signaling. In fact, we showed that C3G promoted actin polymerization during spreading on CRP-XL, but not on fibrinogen. C3G did this by modulating Rac1 activity and by interacting with proteins involved in actin remodeling during lamellipodia formation, such as WAVE2, Arp2/3, Abi1 and VASP [60]. Notably, this function of C3G is independent of Rap1, despite the described role of Rap1b in platelet spreading [61] via Rac1 [25] (Fig. 8). Based on that, it is reasonable to speculate that C3G could modulate Rac1 activity through interaction with proteins harboring SH3 domains, such as c-Cbl or Vav1. In fact, recent findings from our group demonstrated a functional relationship between C3G and c-Cbl in platelets [6], and a role for c-Cbl in lamellipodia formation through a pathway including Vav, PI3K, Crk and Rac1 has been described [62]. In addition, Vav1 plays a role in platelet spreading on collagen but not on fibrinogen [63, 64], and we have documented the interaction of C3G with c-Cbl and Vav1/2 in K562 cells [65]. Hence, our results, supported by the literature, suggest that C3G would regulate lamellipodia formation through a c-Cbl/Crk/Vav1/Rac1 pathway.

Furthermore, our data suggest that C3G, which is known to bind actin [19, 66, 67], might modulate actin polymerization by interacting with actin monomers, facilitating their incorporation into F-actin. Consequently, overexpression of C3G would increase actin polymerization, while its absence might lead to actin depolymerization. Alternatively, C3G, through its binding to *F*-actin, could potentially prevent actin depolymerization. All of this supports a role for C3G in regulating actin dynamics in platelets.

Consistent with the role of C3G in modulating platelet secretion, we showed that C3G absence induced increased release of coagulation factors, while these were retained in tgC3G platelets, similarly to what was observed for angiogenic factors [6, 7]. However, C3G-KO platelets exhibited defective PS translocation to the PM and, consequently, decreased thrombin generation, in line with their impaired thrombus formation [4]. This suggests that the increased secretion of coagulation factors by these platelets is likely a consequence of the altered secretory mechanism described above, and not a compensatory mechanism. Accordingly, C3G-KO platelets presented a significant delay in clot retraction, further supporting a role for C3G in outside-in signaling by linking integrin activation to cytoskeletal mechanical forces [68]. No effect of C3G expression on RhoA activation was observed (data not shown), despite the known role of this GTPase in clot retraction [69]. However, clot retraction in platelets can also be regulated by Rac1 [70]. Defective clot retraction was also found in Cbl^−/−^ platelets [71], further supporting the involvement of Cbl in C3G-mediated outside-in signaling in platelets.

In summary, in this work we present evidence on the role of platelet C3G in α-granule exocytosis, spreading and clot retraction by modulating protein complexes involved in vesicle docking, priming and fusion, and in the remodeling of the actin cytoskeleton. Based on these findings and other results from our group, we propose that C3G serves as a crucial regulator of platelet Rap1, working in conjunction with RasGRP2, owing to their potential interdependence (Fig. S1D). Our hypothesis posits that both C3G and RasGRP2 are indispensable for platelet functions. RasGRP2 serves as the primary initiator of the Rap1-integrin αIIbβ3 pathway during hemostasis, responding to rapid calcium transients that follow activation of platelet receptors. Meanwhile, C3G appears to mediate a secondary wave of Rap1 activation, potentially through PKC, as well as other Rap1-independent mechanisms, leading to α-granule secretion and clot retraction.

### Supplementary Information

Below is the link to the electronic supplementary material.

Supplementary file1 (AVI 538 KB)

Supplementary file2 (AVI 622 KB)

Supplementary file3 (AVI 6701 KB)

Supplementary file4 (AVI 17131 KB)

Supplementary file5 (AVI 11142 KB)

Supplementary file6 (AVI 2187 KB)

Supplementary file7 (AVI 4376 KB)

Supplementary file8 (AVI 4376 KB)

Supplementary file9 (TIF 27813 KB)

Supplementary file10 (TIF 24303 KB)

Supplementary file11 (TIF 20518 KB)

Supplementary file12 (TIF 21660 KB)

Supplementary file13 (TIF 13733 KB)

## Data Availability

Data and further details regarding the manuscript will be made available by request to the corresponding author.
