# Supplementary figures and images for: Platelet C3G: a key player in vesicle exocytosis, spreading and clot retraction
Source: Cell Mol Life Sci. 2024 Feb 12;81(1):84. doi: 10.1007/s00018-023-05109-8 (PMC10861696; doi:10.1007/s00018-023-05109-8)

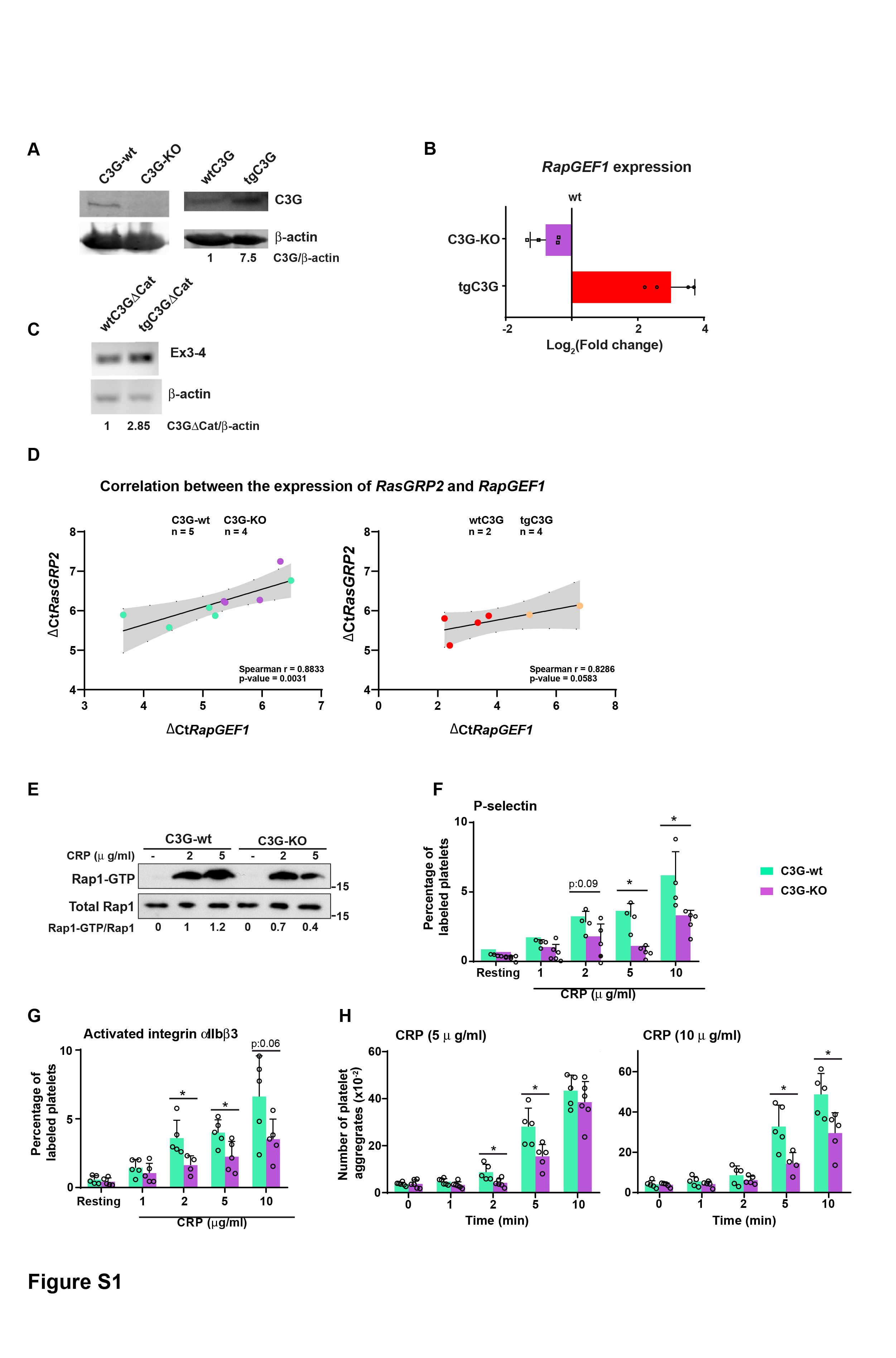

Supplement: Supplementary file 9 — Supplementary file9 (TIF 27813 KB) [file 18_2023_5109_MOESM9_ESM.tif]

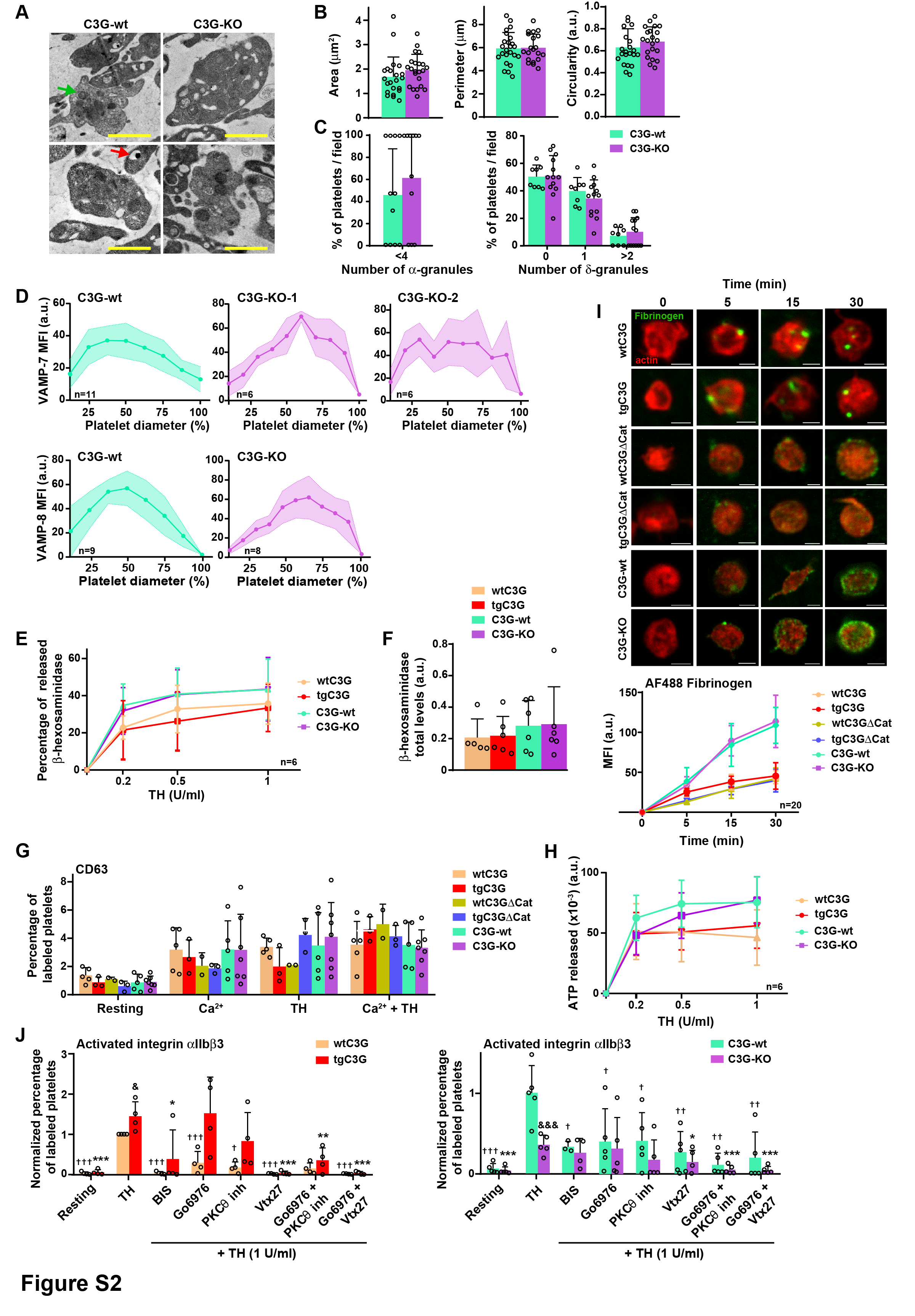

Supplement: Supplementary file 10 — Supplementary file10 (TIF 24303 KB) [file 18_2023_5109_MOESM10_ESM.tif]

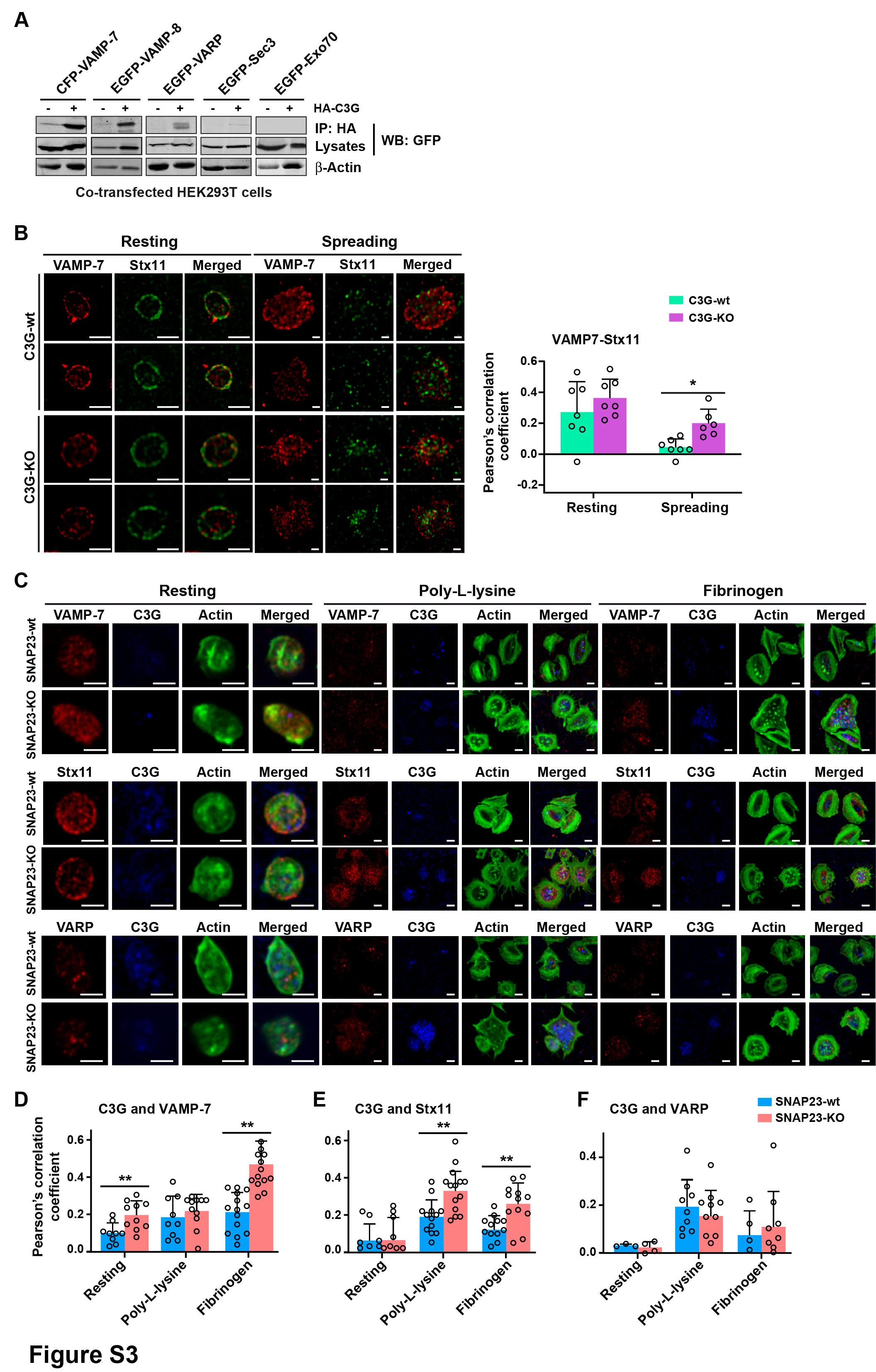

Supplement: Supplementary file 11 — Supplementary file11 (TIF 20518 KB) [file 18_2023_5109_MOESM11_ESM.tif]

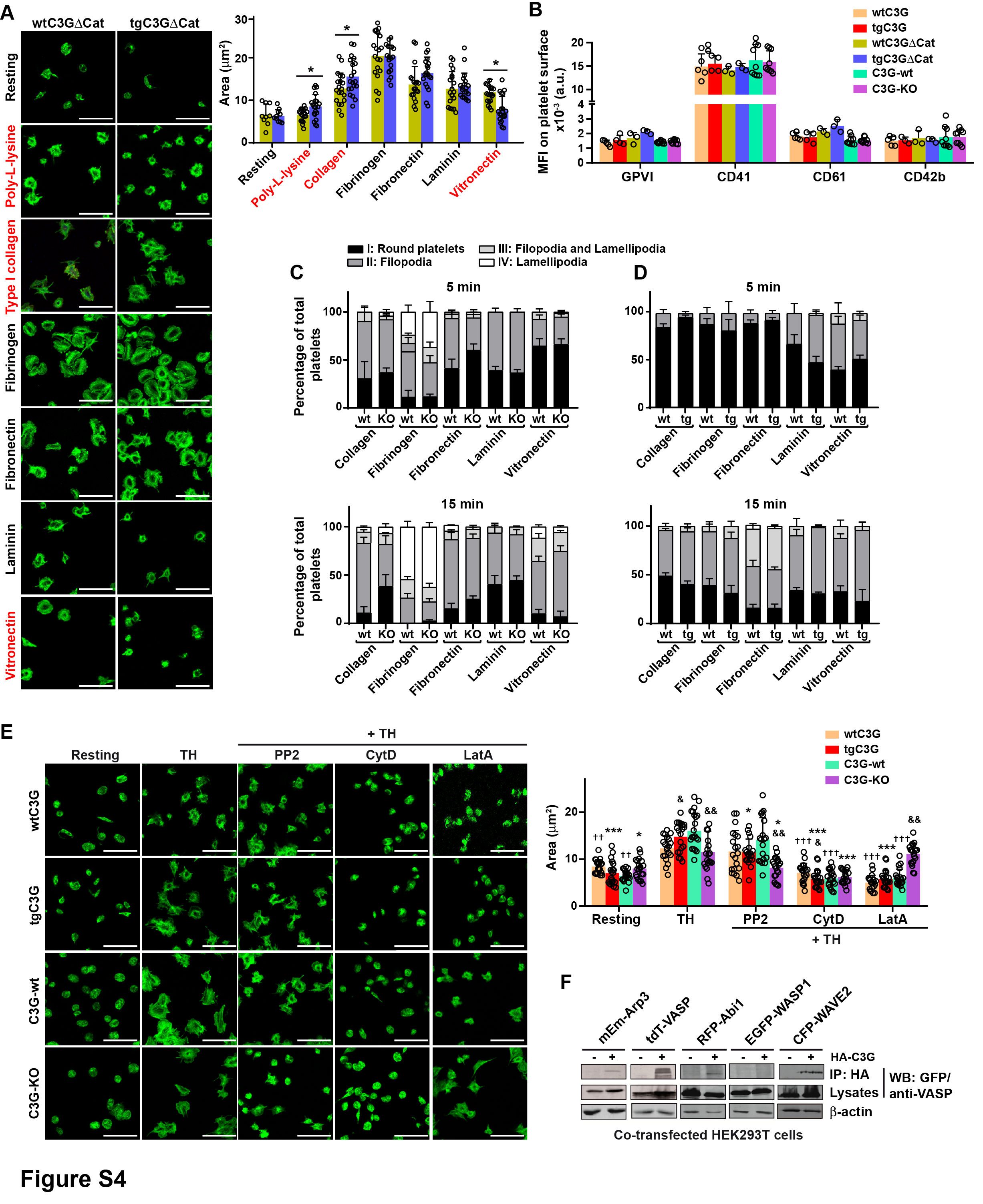

Supplement: Supplementary file 12 — Supplementary file12 (TIF 21660 KB) [file 18_2023_5109_MOESM12_ESM.tif]

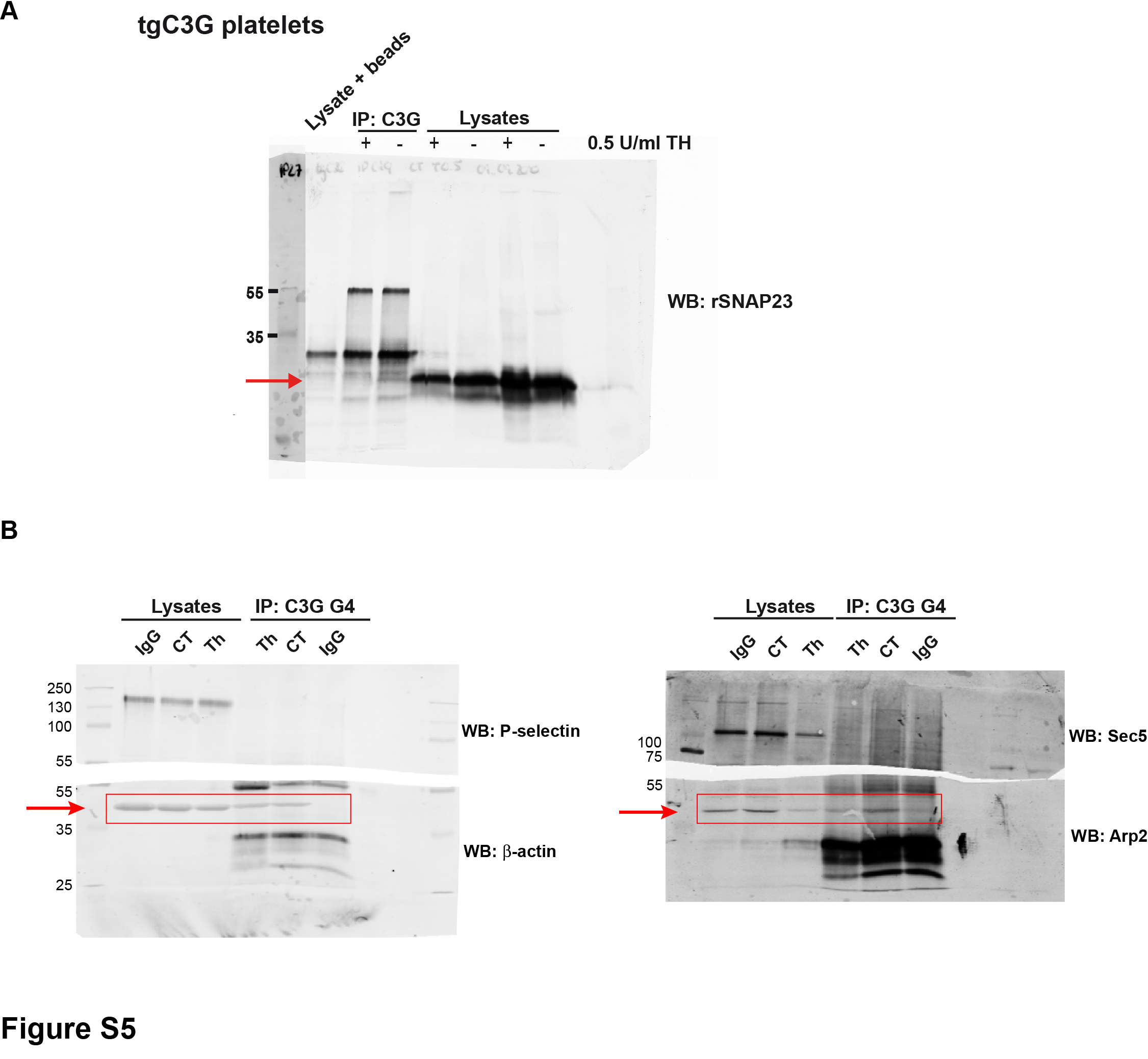

Supplement: Supplementary file 13 — Supplementary file13 (TIF 13733 KB) [file 18_2023_5109_MOESM13_ESM.tif]
